# Involvement of AMP-activated protein kinase and Death Receptor 5 in TRAIL-Berberine-induced apoptosis of cancer cells

**DOI:** 10.1038/s41598-018-23780-x

**Published:** 2018-04-03

**Authors:** Rong Ke, Kanchan Vishnoi, Navin Viswakarma, Sreevidya Santha, Subhasis Das, Ajay Rana, Basabi Rana

**Affiliations:** 1 Department of Surgery, Division of Surgical Oncology, University of Illinois at Chicago, Chicago, IL-60612, USA; 2 University of Illinois Hospital and Health Sciences System Cancer Center, University of Illinois at Chicago, Chicago, IL-60612 USA; 3 Jesse Brown VA Medical Center, Chicago, IL 60612 USA; 4 Present Address: Department of Pharmacology & Therapeutics, Roswell Park Cancer Institute, Buffalo, NY 14263 USA

## Abstract

Our previous studies indicated that combination of Tumor necrosis factor-related apoptosis-inducing ligand (TRAIL) and PPARγ ligand Troglitazone (TZD), can induce significant apoptosis in various TRAIL-resistant prostate and hepatocellular carcinoma (HCC) cells. These also suggested serine/threonine kinase AMP-activated protein kinase (AMPK) to be a mediator of TRAIL-TZD-induced apoptosis. To further validate AMPK’s role in TRAIL sensitization, we determined the apoptotic potential of TRAIL in combination with the natural compound Berberine (BBR), the latter being a potent activator of AMPK. These demonstrated a significant reduction of cell viability and induction of apoptosis (increased cleavage of caspase 3, 8, 9) when treated with TRAIL-BBR combination. This apoptosis is attenuated in cells overexpressing AMPKα-dominant negative (DN) or following AMPKα knockdown, confirming involvement of AMPK. To identify potential downstream mediators involved, an apoptosis RT^2^ PCR array analysis was performed. These showed induction of several genes including TNFRSF10B (expresses DR5) and Harakiri following BBR treatment, which were further validated by qPCR analysis. Furthermore, knocking down DR5 expression significantly attenuated TRAIL-BBR-induced apoptosis, suggesting DR5 to be a mediator of this apoptosis. Our studies indicate that combination of TRAIL and AMPK activator BBR might be an effective means of ameliorating TRAIL-resistance involving DR5 in advanced cancer.

## Introduction

Tumor necrosis factor-related apoptosis-inducing ligand (TRAIL/Apo2L) belongs to the tumor necrosis factor (TNF) ligand superfamily and has been studied extensively for its potential use as a cancer chemotherapeutic agent due to its low toxicity towards normal tissue^1,2^. Despite this proapoptotic activity of TRAIL in transformed tissue, clinical studies with TRAIL or related agents have shown limited efficacy^3^. One of the reasons behind this limitation is attributed to the development of cancer cell resistance towards TRAIL via various mechanisms^4,5^. An understanding of the molecular mechanisms that mediate TRAIL-resistance is critical to identify suitable targets and develop future TRAIL-based therapeutic strategies for advanced cancer.

Resistance towards standard therapeutic regimens and evasion of apoptosis are some of the hallmarks of advanced cancer, which include Sorafenib-resistance in hepatocellular carcinoma (HCC), castration-resistance in prostate cancer (Pca). In an attempt to enhance the apoptotic response of resistant cancer cells, our recent studies have been focused towards developing novel combinatorial strategies to antagonize TRAIL-resistance in cancer cells. These TRAIL combination studies are expected to provide a mechanistic insight towards ameliorating not only TRAIL-resistance but drug-resistance in general. Extensive research over the past decade have revealed some novel combinatorial approaches that can increase cancer cell sensitivity towards TRAIL-induced cytotoxicity. These TRAIL-sensitizing agents include kinase inhibitors^6,7^, Peroxisome Proliferator-activated Receptor γ (PPARγ) agonists^8,9^, histone deacetylase inhibitors^10^ and more^11,12^. Despite their efficacies in increasing TRAIL sensitivity, the detailed signaling mechanism by which this is achieved is still largely unclear and yet to be unraveled. Towards our goal of achieving a mechanistic insight, we have demonstrated that treatment with a combination of TRAIL and PPARγ ligand Troglitazone (TZD) induces profound apoptosis compared to either agent alone^8^. Further insight towards the mechanism involved demonstrated that TRAIL and TZD combination-induced apoptosis was mediated via Adenosine monophosphate-activated protein kinase (AMPK) pathway^13^. These studies showed that while combination of TRAIL-TZD induced potent apoptosis in various cancer cells, this was significantly attenuated following inhibition of AMPK pathway.

AMPK belongs to the family of highly conserved serine threonine kinases and is critically involved in energy homeostasis^14,15^ and in regulating ATP production and consumption. Structurally, it consists of a catalytic subunit (AMPKα), two regulatory subunits (AMPKβ and γ) and their multiple isoforms giving rise to various heterotrimeric compositions. AMPK can be activated via two mechanisms: (i) increase in cellular AMP levels leading to allosteric activation and (ii) via phosphorylation of Thr^172^ residue in the activation loop of the α-subunit by LKB1, CAMKKβ, TAK1 or MLK3^16–19^. Via regulating its downstream targets, AMPK can control various cellular and biological processes such as metabolism, protein translation, cellular growth, autophagy and cancer^20^. A wide variety of pharmacological and natural activators of AMPK are available which include metformin^21^, phenformin^22^, 5-Aminoimidazole-4-Carboxamide Riboside (AICAR)^23^, thiazolidinediones^24^, berberine (BBR)^25^, resveratrol^26^ and are being studied towards their role as anticancer agents.

Based on the facts that the natural compound BBR can activate AMPK and that TRAIL-TZD-induced apoptosis involved AMPK pathway, we postulated that a combination of TRAIL and BBR might also sensitize cells towards apoptosis involving AMPK axis. Our results indicate a significant potentiation of apoptosis when treated with TRAIL and BBR combination, which was also mediated via AMPK. An apoptosis specific RT^2^ Profiler PCR Array performed following BBR treatment to identify potential downstream effectors showed a significant increase in the expression of several genes including Caspase 5, GADD45A, Harakiri, TNFRSF10B (for DR5), TNFSF8 and downregulation of TNFRSF1B. Knockdown studies designed with DR4 or DR5 specific siRNA indicated that TRAIL-BBR-induced apoptosis is mediated predominantly by DR5. These promising results demonstrate the potential to use BBR in ameliorating TRAIL-resistance in advanced forms of cancer and sheds light towards its clinical importance, since no negative side effects of BBR in human is known so far.

## Results

### Effect of treatment with TRAIL and BBR alone or in combination on cancer cell viability

**Figure 1:**
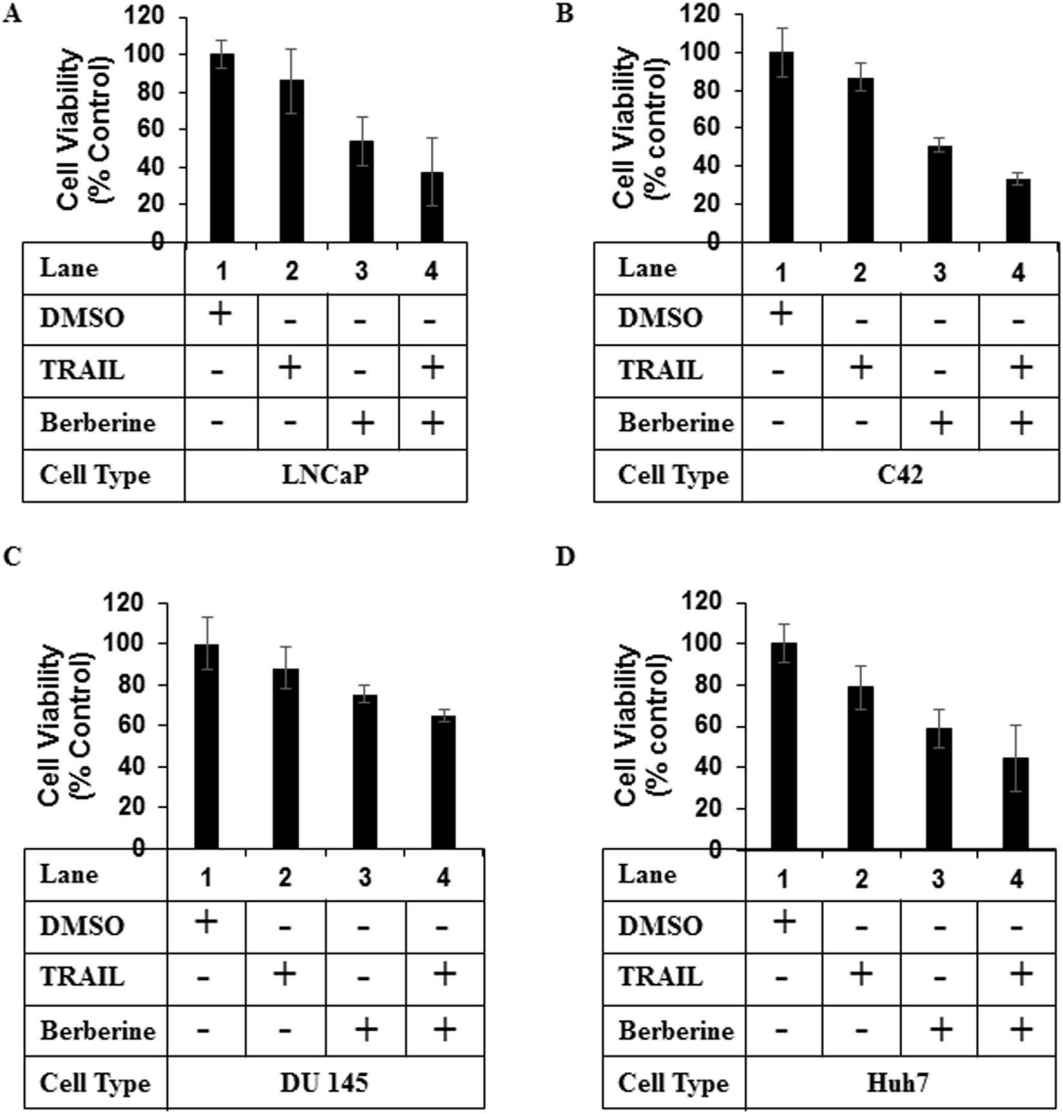
Treatment with a combination of TRAIL and BBR reduces cancer cell viability. (**A**) LNCaP (**B**) C42 (**C**) DU 145 and (**D**) Huh7 cells were treated with DMSO (as vehicle) or TRAIL (100ng/ml) or BBR (50 µM) alone or in combination for 12–16 hrs and subjected to MTT assay. The results were expressed as percentage of control considering the vehicle-treated values as 100%. Each treatment was performed in triplicate and each experiment was repeated at least two times. The data represent the mean ± S.D. of two independent experiments.

In earlier studies, we have demonstrated that combination of TRAIL along with TZD can induce potent apoptosis in various resistant cancer cells, an effect that involved AMPK^13^. In order to determine whether other activators of AMPK can also mediate similar apoptotic responses in cancer cells, in the current studies we utilized the natural compound BBR, which is a known activator of AMPK^25^. Treatment of various Pca cells as well as HCC cells with a combination of TRAIL and BBR significantly reduced cell viability and at a higher potency compared to either agent alone (Fig. 1A–D and Supplementary Fig. S1).

### Effect of TRAIL-BBR combination on the apoptotic potential of cancer cells

**Figure 2:**
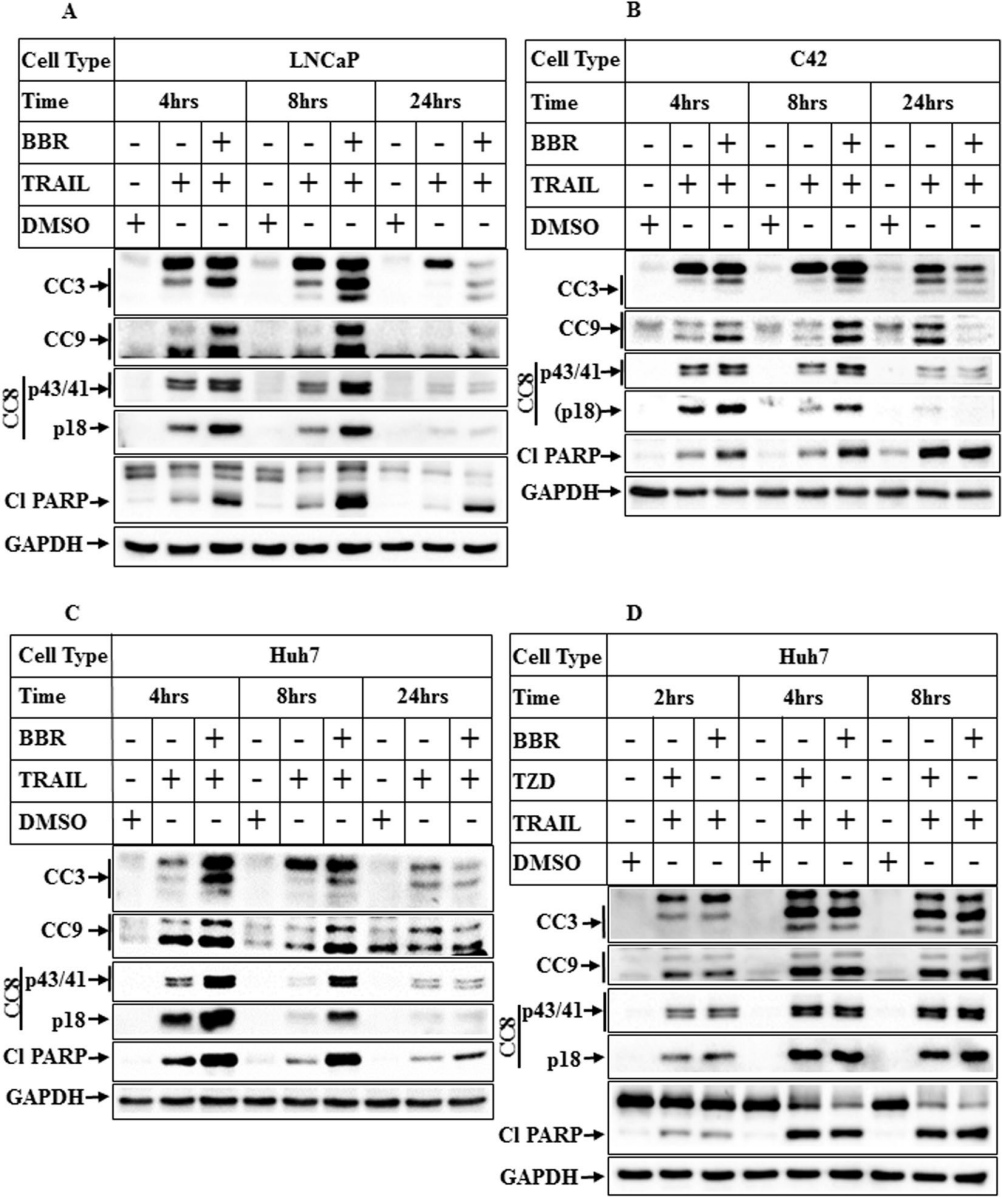
Combinatorial treatment with TRAIL and BBR induces potent apoptosis in cancer cells. Confluent populations of (**A**) LNCaP (**B**) C42 or (**C**) Huh7 cells were treated with DMSO or TRAIL (100ng/ml) alone or in combination with BBR (50 µM) for the indicated periods of time. Cells were harvested at the end of treatment and analyzed by Western blots with the indicated antibodies specific for apoptosis and GAPDH was used as control. (**D**) Huh7 cells were treated for the indicated periods of time with DMSO or a combination of TRAIL and TZD or TRAIL and BBR. Western blot analyses were performed with the indicated antibodies. [CC3, 8, 9: Cleaved Caspase 3, 8, 9; Cl PARP: Cleaved Poly (ADP-ribose) Polymerase]. For all western blots (**A**–**D**), the bands from adjacent lanes were cropped from the same blot (full-length uncropped blots are included in Supplementary Fig. S4).

To determine whether the reduction of cell survival following treatment with TRAIL-BBR combination was due to increased apoptosis, western blot analyses were performed following treatment with these agents for different periods of time. These showed a significant increase in cleavage of caspases 8, 9, 3 and PARP (indicative of apoptosis) when treated with the combination compared to TRAIL alone (Fig. 2A–C and Supplementary Fig. S2). To compare whether the degree of this apoptosis was comparable to that induced by TRAIL-TZD combination, Huh7 cells were treated with vehicle or TRAIL in combination with TZD or BBR for various lengths of time. These showed that TRAIL-BBR-combination was equally potent towards apoptosis induction when compared with TRAIL-TZD combination (Fig. 2D).

### Effect of increasing concentration of BBR on TRAIL-induced apoptosis

**Figure 3:**
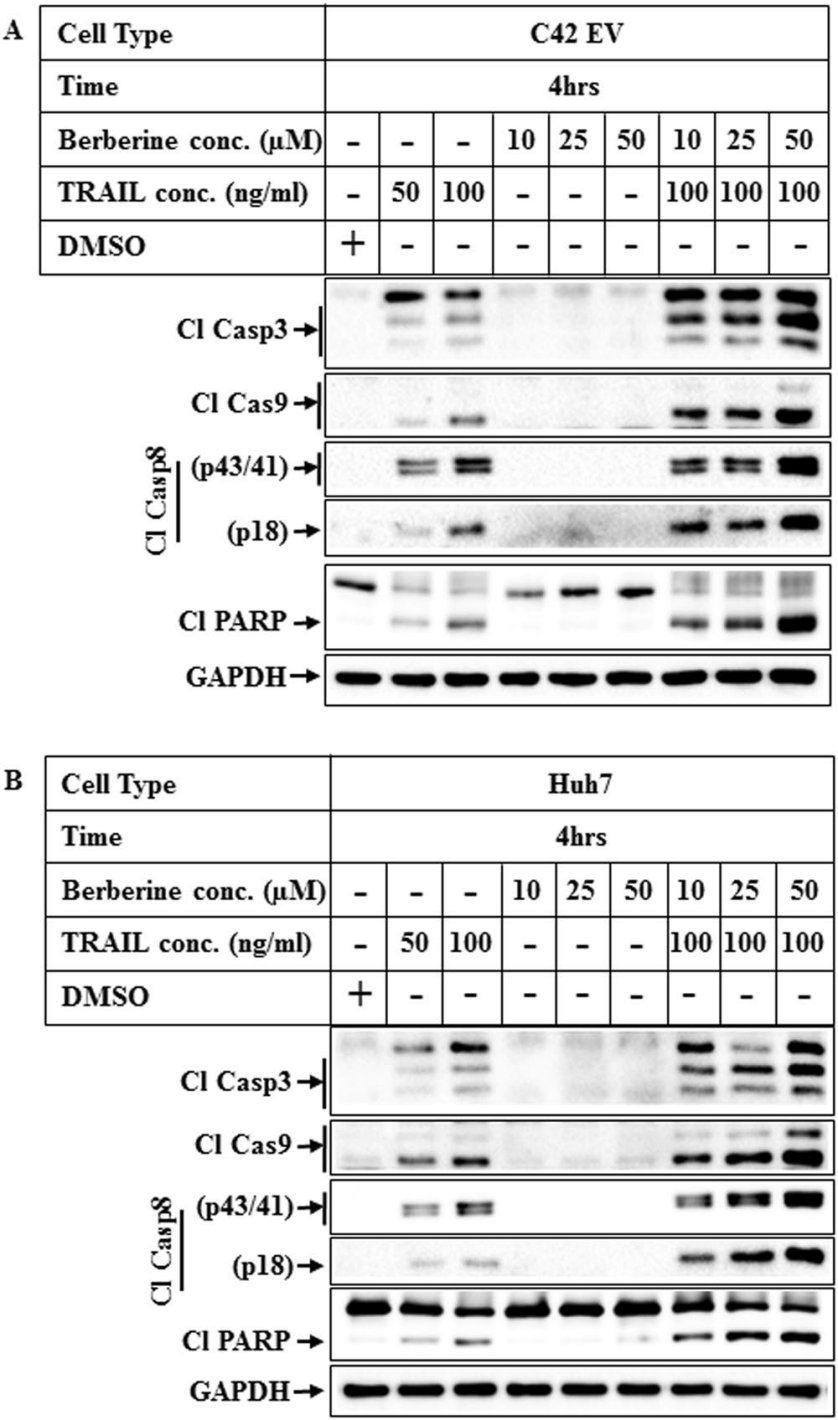
Effect of increasing concentrations of TRAIL and BBR on apoptosis. (**A**) C42 EV (overexpressing empty vector) and (**B**) Huh7 cells were treated for 4 hrs with DMSO or increasing concentrations of TRAIL alone (lanes 2, 3) or BBR alone (lanes 4–6) or a combination of TRAIL (100ng/ml) and increasing concentrations of BBR (lanes 7–9). Western blot analyses were performed with the indicated antibodies. For all western blots (**A**,**B**), the bands from adjacent lanes were cropped from the same blot (full-length uncropped blots are included in Supplementary Fig. S5).

In order to determine the optimal concentrations of TRAIL and BBR needed to increase apoptosis significantly, cells were treated with increasing concentrations of TRAIL or BBR alone or in combination. These showed that although TRAIL alone can induce some degree of apoptosis (Fig. 3A,B, lanes 2–3), this was significantly potentiated when combined with BBR which was maximum at 25–50 µM concentrations (lanes 7–9). Based on these results, the apoptosis studies described here were carried out with a combination of 100ng/ml TRAIL and 50 µM BBR.

### Involvement of AMPK in TRAIL-BBR combination-induced apoptosis

**Figure 4:**
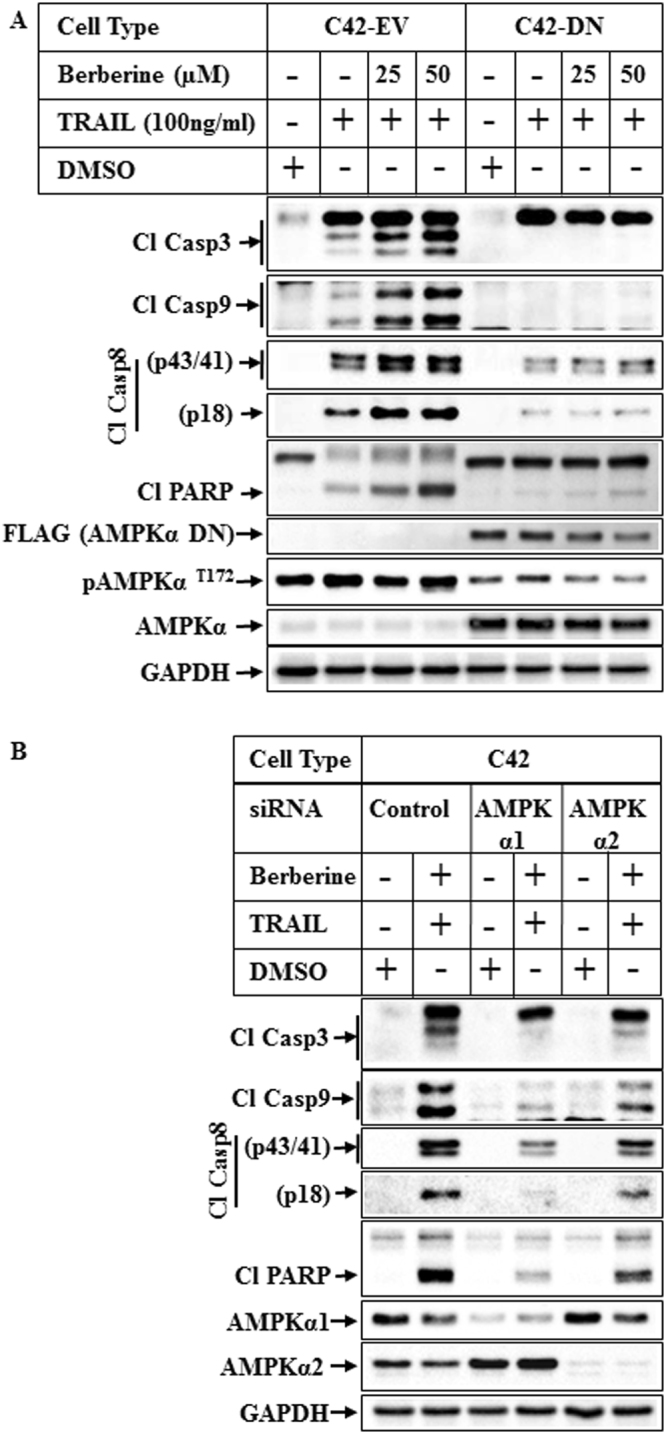
Effect of AMPKα inhibition on TRAIL-BBR-induced apoptosis. (**A**) C42 cells overexpressing empty vector (C42-EV) or FLAG epitope-tagged dominant negative AMPKα1 vector (C42-DN) were treated with DMSO or TRAIL alone or TRAIL in combination with increasing concentrations of BBR for 8 hrs. The samples were analyzed by Western blot analyses with the indicated antibodies. (**B**) C42 cells were transiently transfected with 50 nM of either control siRNA (lanes 1 & 2), or AMPKα1 siRNA (lanes 3 & 4) or AMPKα2 siRNA (lanes 5 & 6) for 72 hrs and then treated with DMSO or a combination of TRAIL and BBR for 8 hrs. Western blot analysis was performed utilizing the antibodies indicated. For all western blots (**A**,**B**), the bands from adjacent lanes were cropped from the same blot (full-length uncropped blots are included in Supplementary Fig. S6).

Since BBR is a known activator of AMPK, we next determined whether TRAIL-BBR-induced apoptosis was mediated by AMPK. Utilizing Pca cells overexpressing either empty vector (C42-EV) or dominant negative AMPKα (C42-DN)^27^, we compared the apoptotic potential of TRAIL-BBR combination in these two cell types. These results demonstrated an increase in the apoptosis profile in the C42-EV cells when treated with a combination of TRAIL-BBR, which was significantly attenuated in the C42-DN cells (Fig. 4A). To confirm the role of AMPK further, TRAIL-BBR-induced apoptosis was carried out following knocking down of endogenous AMPKα1 or α2. These results demonstrated that while knockdown of either AMPKα1 or α2 can attenuate TRAIL-BBR-induced apoptosis, the effects were predominantly mediated by AMPK α1 (Fig. 4B).

### Analysis of BBR-induced apoptotic gene expression using RT^2^ Profiler PCR Array

**Figure 5:**
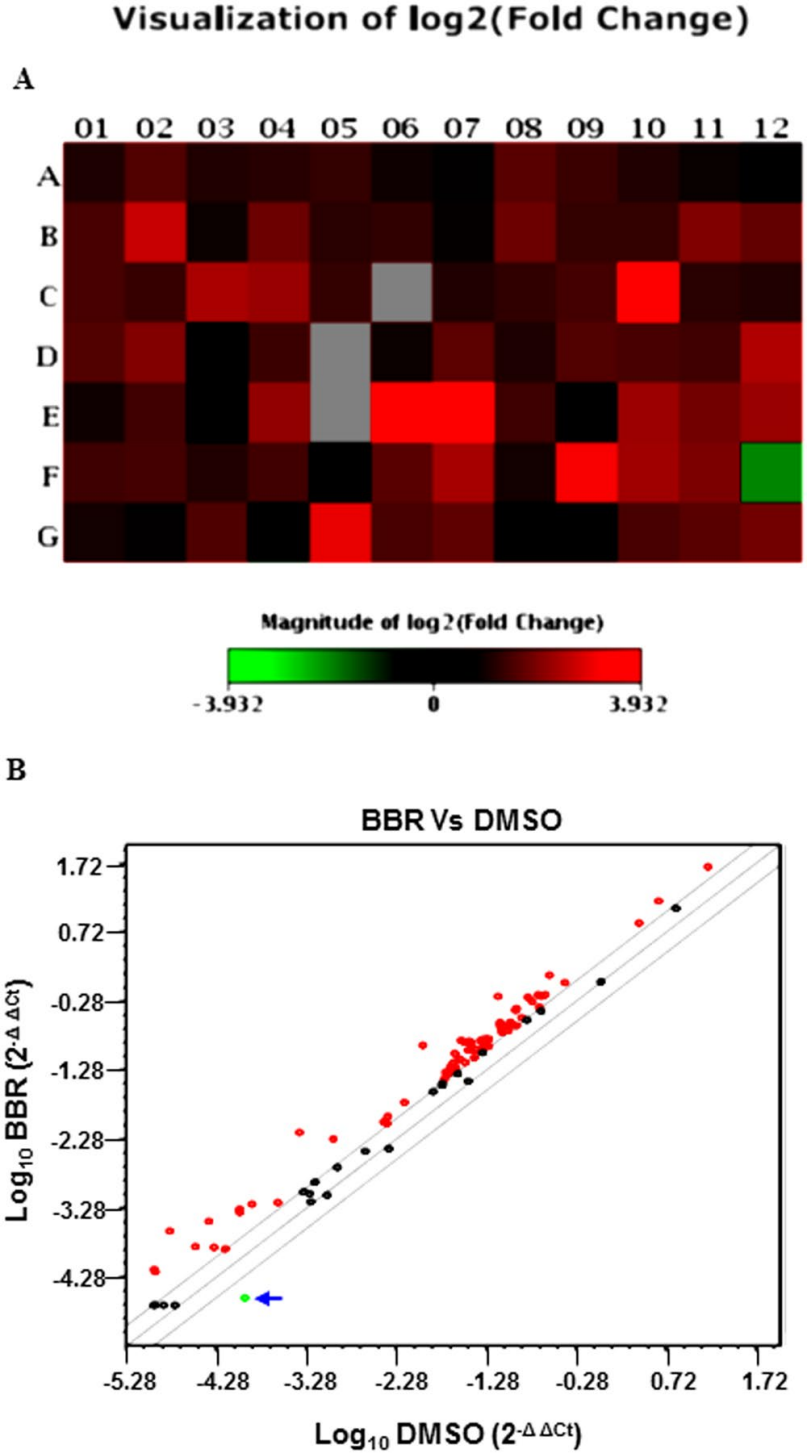
RT^2^ Profiler PCR Array analysis of human apoptotic gene expression. Total RNA was extracted from LNCaP cells treated with DMSO or BBR (50 µM) for 16 hrs and subjected to cDNA synthesis and analyzed by human apoptosis PCR Array (PAHS-012Z). Fold changes >2.0 and a *P*-value < 0.05 were considered to be significant variations. The data represent the mean of two independent experiments. (**A**) Shows the heat map with the upregulated genes marked in red and the downregulated gene marked in green and (**B**) includes an overview of scatter plot on expression levels of 84 genes. Red dots indicate genes upregulated and the Green dot (marked by an arrow) indicates the only gene downregulated in BBR-treated cells. The central line indicates genes that were unchanged with boundaries representing the two-fold regulation cut-off. Genes Highly Induced: **(C10) CASP5**, Caspase 5, apoptosis-related cysteine peptidase; (**E06) GADD45A**, Growth arrest and DNA-damage-inducible, alpha; **(E07) HRK**, Harakiri, BCL2 interacting protein (contains only BH3 domain); **(F09) TNFRSF10B**, Tumor necrosis factor receptor superfamily, member 10b**; (G05) TNFSF8**, Tumor necrosis factor (ligand) superfamily, member 8; Gene Downregulated: **(F12) TNFRSF1B**, Tumor necrosis factor receptor superfamily, member 1B.

The studies above indicated that BBR might modulate the apoptotic pathway to potentiate the effects of TRAIL. To determine the effect of BBR on apoptotic gene expression, cells treated with vehicle or BBR were subjected to RNA extraction, cDNA synthesis and RT^2^ Profiler PCR Array analysis (using PAHS-012Z) as per manufacturer’s instructions. Those genes that showed a fold change of >2 (and a *P*-value of <0.05) were considered significant and are shown in Fig. 5A,B and Supplementary Fig. S3. The genes that were highly induced included a 15 fold induction of Caspase 5, apoptosis-related cysteine peptidase (CASP5), 11 fold induction of Growth arrest and DNA-damage-inducible, alpha (GADD45A), >14 fold induction of Harakiri, BCL2 interacting protein (contains only BH3 domain) (HRK), >8 fold induction of Tumor necrosis factor receptor superfamily, member 10b (TNFRSF10B), >7 fold induction of Tumor necrosis factor (ligand) superfamily, member 8 (TNFSF8) (Supplementary Fig. S3). The only gene that was down regulated 4 fold was Tumor necrosis factor receptor superfamily, member 1B (TNFRSF1B).

### Validation of BBR-induced changes of Harakiri and DR5 gene expression by qPCR

**Figure 6:**
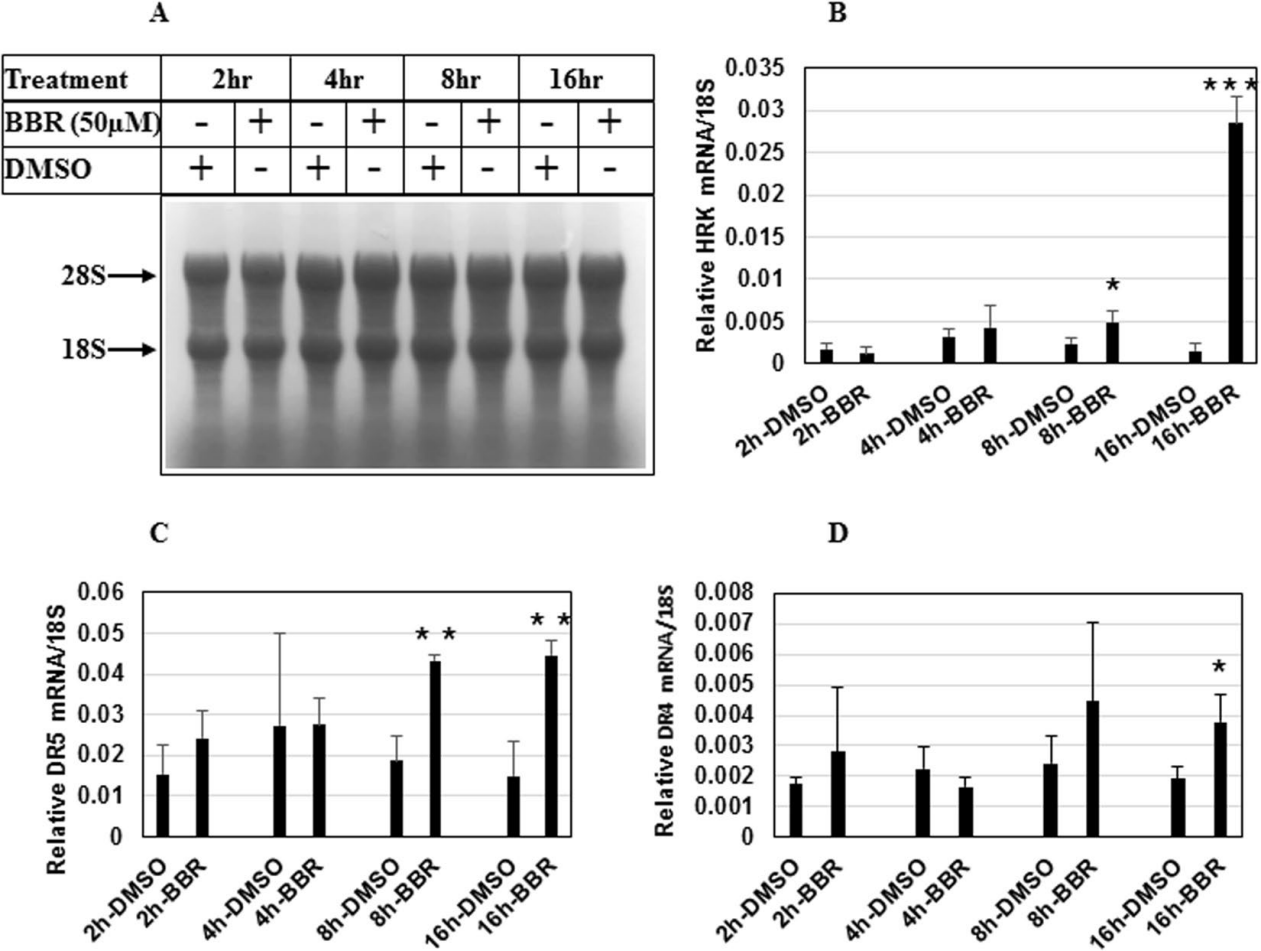
Validation of BBR-induced changes of gene expression by qPCR. (**A**) Agarose gel showing equal loading of RNA extracted from LNCaP cells treated with vehicle (DMSO) or BBR (50 µM) for different lengths of time. Changes in gene expression of HRK (**B**), DR5 (**C**) and DR4 (**D**) following BBR treatment were validated by qPCR analysis. The experiment was repeated twice and data represent the mean ± S.D. of three independent PCR reactions. Significant differences were determined by *t*-test and indicated: ns, *P* > 0.05, *P ≤ 0.05, **P ≤ 0.01, ***P ≤ 0.001.

To validate the changes in apoptosis-specific gene expressions obtained in RT^2^ Profiler PCR Array analysis, qPCR analyses were performed with RNA extracted from cells treated with BBR for various lengths of time using primers for Harakiri, Death receptor 4 (DR4) and DR5 as listed (Supplementary Table S1) with 18S rRNA as control. These showed a significant induction of HRK and DR5 mRNA expressions when treated with BBR, while DR4 changes were more modest (Fig. 6A–D, Supplementary Table S2).

### Involvement of DR5 in TRAIL-BBR combination-induced apoptosis

**Figure 7:**
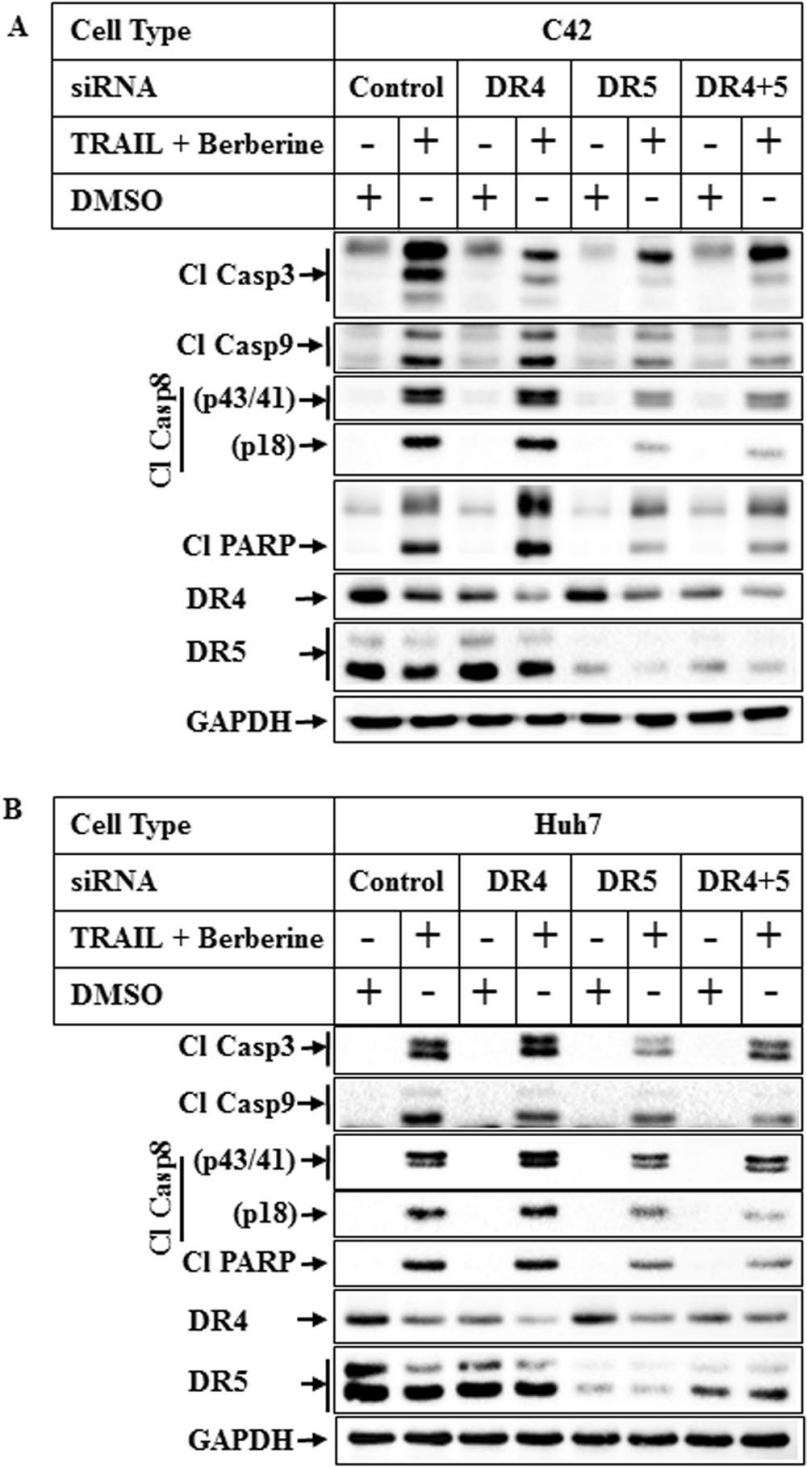
Role of DR4 and DR5 in mediating TRAIL-BBR-induced apoptosis. Subconfluent populations of C42 **(A**) or Huh7 (**B**) cells were transiently transfected with 50 nM of either control siRNA (lanes 1 & 2), or DR4 siRNA (lanes 3 & 4) or DR5 siRNA (lanes 5 & 6) or both DR4 and DR5 siRNA (lanes 7 & 8) followed by treatment with DMSO or a combination of TRAIL (100ng/ml) and BBR (50 µM) for 6 hrs. The samples were analyzed by Western blots with the indicated antibodies. For all western blots (**A,B**), the bands from adjacent lanes were cropped from the same blot (full-length uncropped blots are included in Supplementary Fig. S7).

Apoptosis induction via TRAIL involves extrinsic pathway mediated via two receptors DR4 and DR5^5^. Since BBR showed an induction of DR5 gene expression we hypothesized that this apoptotic pathway might be mediated via DR5. To obtain an insight into the mechanism how TRAIL-BBR mediated the apoptosis pathway, we determined whether this involved the death receptors DR4 or DR5. Endogenous expression of DR4 or DR5 or both were knocked down first followed by treatment with TRAIL-BBR combination. These results showed that cleavage of caspases and PARP (indicating apoptosis) are predominantly mediated by DR5 in cancer cells (Fig. 7A,B), although additional mechanisms are likely involved as well.

## Discussion

Despite a significant amount of research efforts towards developing TRAIL-based cancer therapies, TRAIL receptor agonists have not shown significant efficacy clinically^28,29^. Developing combination therapies targeting pathways that promote TRAIL-resistance is needed to increase TRAIL sensitivity. In earlier studies we have demonstrated that treatment of TRAIL-resistant cells with a combination of TRAIL and PPARγ agonist TZD can increase their apoptotic potential and activate caspase-induced apoptosis^8^. In fact, various TRAIL combination studies have been reported by different investigators that show promise towards ameliorating TRAIL-resistance. These include inhibitors of signaling pathways^6,7^, PPARγ agonists^8,9^, histone deacetylase inhibitors^10^ and more^11,12^. In order to develop these drug combinations as effective cancer therapies, it is however, necessary to have a clear understanding of how these function. To elucidate the mechanism involved, in more recent studies we have demonstrated that AMPK pathway is involved in mediating TRAIL-TZD combination-induced apoptosis^13^. These suggested the possibility that combination of TRAIL with various AMPK agonists might be an effective means of targeting TRAIL-resistant cells. This is supported by the recent report that metformin can sensitize triple negative breast cancer cells to TRAIL-induced apoptosis^12^. The authors demonstrated that metformin’s effects were mediated via reducing XIAP expression, which is an antagonist of TRAIL-induced apoptosis. With metformin being a type II diabetic drug and an agonist of AMPK^21^, we hypothesized that combination treatment with other AMPK activators might ameliorate TRAIL-resistance in cancer cells. To address this, in the current studies we explored the apoptotic potential of combining TRAIL and BBR, since BBR is a known activator of AMPK^25^. Although TRAIL and BBR combination has been shown to induce apoptosis in earlier studies^30,31^, a clear mechanistic insight towards the downstream mediators is still unknown. Our results showed that combination of TRAIL with BBR potentiated the apoptotic effects of TRAIL in both HCC and Pca cells. This was associated with a significant reduction of cell viability with combination treatment and an increase in the cleavage of Caspase 8, 9, 3 and PARP, indicative of apoptosis. Utilizing cells overexpressing dominant negative (DN) AMPK, we demonstrated that TRAIL-BBR-induced apoptosis was significantly attenuated in the DN cells as indicated by reduced caspase activation. Furthermore, knocking down endogenous AMPKα1 or α2 expression attenuated TRAIL-BBR-induced apoptosis. These further validated the participation of AMPK in mediating TRAIL-BBR-combination-induced apoptosis.

To elucidate the mechanism by which BBR might be sensitizing the cells towards TRAIL, we performed an apoptosis specific RT^2^ Profiler PCR Array with RNA extracted from cells treated with BBR. These showed a significant increase in the expression of several apoptotic genes including Casp5, GADD45A, Harakiri, TNFRSF10B, TNFSF8 and downregulation of TNFRSF1B. qPCR studies were designed with specific primers to validate the results of PCR Array, which showed that BBR indeed can induce expression of HRK and DR5 genes significantly, while its effects on DR4 gene expression was modest. These results provided a clue on the potential mechanism by which BBR might promote TRAIL-induced apoptosis and suggested that modulation of DR5 expression might be one of them. To determine which of the TRAIL receptors (DR4 or DR5) mediated TRAIL-BBR-induced apoptosis, we knocked down these receptor expressions either alone or in combination. Interestingly, knocking down DR5 expression significantly attenuated TRAIL-BBR-induced apoptosis suggesting that this apoptotic cascade is predominantly mediated via DR5, although the involvement of additional mechanisms are also possible. Considering AMPK’s profound role in regulating the metabolic pathways and antagonizing mammalian target of rapamycin (mTOR) signaling axis, it is conceivable that these downstream signaling molecules might also play a role in increasing TRAIL sensitivity.

It is however, unclear how BBR induces DR5 gene expression and whether this involves AMPK activity. It has been reported that pharmacological inhibition of mTOR pathway can induce DR5 expression via transcription factor CHOP^32^. Since AMPK is known to antagonize the mTOR pathway^33^, one possibility is that BBR-induced activation of AMPK might antagonize mTOR signaling leading to increased DR5 expression. In addition, DR5 can be induced via p53 pathway^34^. Since BBR can also induce p53 expression^35^, the second possibility is the involvement of p53 in DR5 induction. In fact, the LNCaP cells used in our studies (Figs 5,6 and Supplementary Fig. S3) express wild type p53 as reported earlier^36,37^. The role of p53 can be determined by comparing BBR-induced DR5 expression in p53 wild type and p53 null cancer cells. Studies are currently underway to address these possibilities. In addition, further studies are needed to determine whether any of the other molecules identified in our PCR Array are also involved in mediating TRAIL-BBR effects. In conclusion, our results suggest that treatment with a combination of TRAIL and AMPK agonist BBR can induce potent apoptosis mediated via AMPK and involving DR5. Due to limited availability of therapeutic options to treat resistant forms of cancer, combinatorial treatment with TRAIL and BBR or other AMPK agonists might be an effective means of ameliorating therapeutic resistance.

## Materials and Methods

### Reagents and Antibodies

RPMI tissue culture media, Lipofectamine 2000 were purchased from Invitrogen (Carlsbad, CA); Berberine (catalogue number B3251, purity ≥98%) was from Sigma (St. Louis, MO), Troglitazone, TRAIL were purchased from EMD Biosciences (Gibbstown, NJ). The antibodies utilized were obtained from the following sources: poly (ADP-ribose) polymerase (PARP), caspase-3, caspase 8, caspase 9, pAMPK^T172^, total AMPK, AMPKα1 and α2, DR4, DR5 from Cell Signaling Technologies (Danvers, MA), FLAG from Sigma (St. Louis, MO), GAPDH from Ambion Inc. (Austin, TX)**;** TRIzol reagent from Invitrogen, (Carlsbad, CA), RT^2^ PCR Profiler PCR Array PAHS-012Z (Cat # 330231) was from Qiagen (Valencia, CA).

### Cell Culture

Human Prostate cancer cells (LNCaP, DU 145) were obtained from ATCC and C42, C42-EV and C42-DN cells were obtained as described^13,27^ and maintained in RPMI-1640 media supplemented with 10% FBS, 100 IU/ml penicillin, and 100 μg/ml streptomycin. Human HCC cells (Huh7) were obtained as described^8^ and maintained in DMEM/F12 media with 10% FBS and 1% Pen/Strep. In TRAIL and BBR or TRAIL-TZD experiments, cells were treated with 100 ng/ml TRAIL alone or in combination with 50 µM Berberine or 50 μM TZD (unless indicated otherwise) for various lengths of time followed by Western blot analysis.

### MTT Assay

MTT assays were performed as described previously^13^. Briefly, cells were plated at a density of 10,000 cells/well in 96-well plates. Following overnight attachment, they were treated in serum-containing medium with DMSO (as vehicle) or TRAIL (100 ng/ml) or BBR (50 µM) alone or in combination for 12 or 16 hrs. At the end of each treatment, the cells were incubated with 0.5 mg/ml MTT solution for 3–4 hrs at 37 °C, followed by incubation with DMSO for another 30 mins at 37 °C and measurement of absorbance at 570 nm in a microtiter plate reader.

### RNA Isolation and RT2 Profiler PCR Array

Total RNA was extracted from LNCaP cells treated with vehicle (DMSO) or Berberine (50 µM) for 16 hrs using TRIzol reagent and the quality of RNA was checked by 1% Agarose gel electrophoresis. Equal amounts of total RNA (2.5 µg) was then reverse transcribed to cDNA using RT^2^ First Strand kit (Qiagen) and Real-Time PCR was performed using SYBR Green Mastermix with human apoptosis RT^2^ Profiler PCR Array (PAHS-012Z, Qiagen) on StepOne Plus machine as per manufacturer’s protocol. Fold changes in expression of mRNA transcripts in BBR-treated compared to vehicle-treated groups were analyzed using SABiosciences webportal software (http://www.sabiosciences.com/pcrarraydataanalysis.php) and *P*-values were calculated using Student’s *t*-test. A *P*-value < 0.05 and a fold change >2.0 were considered to be a significant variation.

### Quantitative Polymerase Chain Reaction (qPCR) Analysis

qPCR analysis was performed as described earlier^38^. Briefly, total RNA was extracted from LNCaP cells treated with DMSO or BBR for various lengths of time and the integrity of 18 S and 28 S ribosomal RNA assessed by gel electrophoresis to confirm RNA quality. cDNA synthesis was performed using Superscript III First-Strand Synthesis System kit (Invitrogen, Carlsbad, CA) and amplified using SYBR Green PCR Master Mix (Applied Biosystems) in ABI StepOnePlus detection system (Applied Biosystems). The PCR cycling condition was set as: 50 °C for 2 min followed by an initial denaturation step at 95 °C for 10 min, 40 cycles at 90 °C for 10 s, 60 °C for 30 s finally subjecting to melting temperature to check amplification curve. The relative changes in gene expression were estimated using the 2−ΔΔCt method using 18 S rRNA as a housekeeping gene. The lists of primers used are included in Supplementary Table S1.

### Small Interference RNA (siRNA)

siRNA smart pool against hAMPKα1 (Cat # L-005027-00), hAMPKα2 (Cat # L-005361-00), DR4 (cat # L-008090-00), DR5 (cat # L-004448-00) were purchased from Dharmacon (Lafayette, CO). A negative control siRNA from Ambion Inc. (Austin, TX) was used as control siRNA. siRNA transfection was performed using Lipofectamine 2000 as per the manufacturer’s instructions and as described previously^13^. Briefly, subconfluent cells plated in 35 mm plates were transfected with 50 nM of either control siRNA or target siRNA for 24 hrs followed by recovery in serum containing medium. The transfected cells were treated after 72 hrs of transfection with either DMSO or a combination of TRAIL and BBR for an additional 8 hrs followed by western blot analysis.

### Western Blot Analysis

Western blot analysis was performed utilizing procedures described previously^39,40^. Briefly, equal amounts of total cell extracts were fractionated by SDS-PAGE, transferred to PVDF membranes, and subjected to Western blot analysis utilizing various antibodies.

### Statistical analyses

Student’s t-test was performed and expressed as *P ≤ 0.05, **P ≤ 0.01, ***P ≤ 0.001, ns: *P* > 0.05 not significant.

### Data availability

The datasets generated during and/or analysed during the current study are available from the corresponding author on reasonable request.

## Electronic supplementary material

Supplementary Information
