# Supplementary material for: Involvement of AMP-activated protein kinase and Death Receptor 5 in TRAIL-Berberine-induced apoptosis of cancer cells
Source: Sci Rep. 2018 Apr 3;8:5521. doi: 10.1038/s41598-018-23780-x (PMC5882856; doi:10.1038/s41598-018-23780-x)

Supplementary information

Involvement of AMP-activated protein kinase and Death Receptor 5 in TRAIL-Berberine-induced apoptosis of cancer cells

Rong Ke[1], Kanchan Vishnoi[1, §], Navin Viswakarma[1, §], Sreevidya Santha [1, 4, §], Subhasis Das[1], Ajay Rana[1, 2, 3], Basabi Rana[1, 2, 3*]

[1]Department of Surgery, Division of Surgical Oncology, University of Illinois at Chicago, Chicago, IL-60612, USA
[2]University of Illinois Hospital and Health Sciences System Cancer Center, University of Illinois at Chicago, Chicago, IL-60612, USA
[3] Jesse Brown VA Medical Center, Chicago, IL-60612, USA

[4]Present address: Department of Pharmacology & Therapeutics, Roswell Park Cancer Institute, Buffalo, NY 14263

§: Equal Contribution

*To whom correspondence should be addressed: Clinical Sciences Building, MC 958, Rm. 638, University of Illinois at Chicago, 840 S. Wood Street, Chicago IL 60612, USA. Tel.: 312-996-1078; Fax: 312-996-9365: E-mail: basrana@uic.edu.

Supplementary Data:

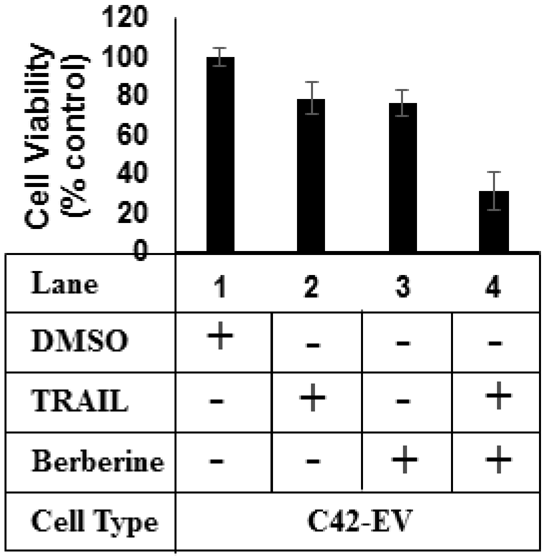

Lane	1	2	3	4
DMSO	+	-	-	-
TRAIL	-	+	-	+
Berberine	-	-	+	+
Cell Type	C42-EV			

Supplementary Figure S1: ***Effect of treatment with a combination of TRAIL and BBR on cancer cell viability*:** C42-EV cells were treated with DMSO or TRAIL (100ng/ml) or BBR (50 μM) alone or in combination for 16hrs and subjected to MTT assay. The results were expressed as percentage of control considering the vehicle-treated values as 100%. Each treatment was performed in triplicate and each experiment was repeated at least two times. The data represent the mean ± S.D. of two independent experiments.

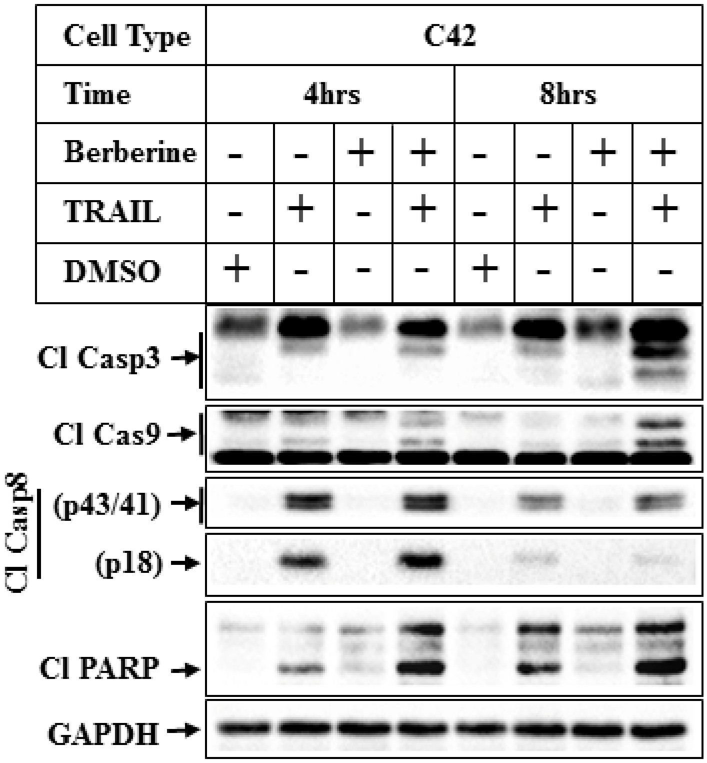

Supplementary Figure S2: ***Effect of treatment with TRAIL and BBR alone or in combination on cancer cell apoptosis:*** Confluent populations of C42 cells were treated with DMSO or TRAIL or BBR alone or in combination for the indicated periods of time followed by Western blot analysis.

Position	Gene Symbol	Fold Regulation	Position	Gene Symbol	Fold Regulation
A01	ABL1	2.05	D08	CIDEA	2.0498
A02	AIFM1	2.886	D09	CIDEB	2.9099
A03	AKT1	2.1168	D10	CRADD	2.7543
A04	APAF1	2.1826	D11	CYCS	2.6164
A05	BAD	2.4048	D12	DAPK1	5.3724
A08	BAK1	3.039	E02	DIABLO	2.6221
A09	BAX	2.51	E04	FAS	4.5068
A10	BCL10	2.123	**E06**	**GADD45A**	**11.3551**
B01	BCL2L1	2.757	**E07**	**HRK**	**14.6908**
B02	BCL2L10	6.3409	E08	IGF1R	2.5768
B04	BCL2L2	3.4564	E10	LTA	4.7468
B05	BFAR	2.2731	E11	LTBR	3.6082
B06	BID	2.3564	E12	MCL1	4.7006
B08	BIRC2	3.4632	F01	NAIP	2.5623
B09	BIRC3	2.4195	F02	NFKB1	2.6582
B10	BIRC5	2.3974	F03	NOD1	2.1217
B11	BIRC6	3.9033	F04	NOL3	2.6115
B12	BNIP2	3.2653	F06	RIPK2	3.0439
C01	BNIP3	2.7378	F07	TNF	5.0396
C02	BNIP3L	2.454	**F09**	**TNFRSF10B**	**8.6961**
C03	BRAF	5.1957	F10	TNFRSF11B	4.8171
C04	CASP1	4.6151	F11	TNFRSF1A	3.8417
C05	CASP10	2.4005	G03	TNFRSF9	2.8564
C07	CASP2	2.1163	**G05**	**TNFSF8**	**7.7553**
C08	CASP3	2.3449	G06	TP53	2.7249
C09	CASP4	2.6958	G07	TP53BP2	3.1439
**C10**	**CASP5**	**15.2681**	G10	TRAF2	2.7438
C11	CASP6	2.2372	G11	TRAF3	3.0301
C12	CASP7	2.1169	G12	XIAP	3.6155
D01	CASP8	2.9183	H03	GAPDH	3.4984
D02	CASP9	4.0091	H04	HPRT1	2.0003
D04	CD40	2.5062	H05	RPLP0	3.0532
D07	CFLAR	3.1216	H06	HGDC	5.6257

**F12**	**TNFRSF1B**	**-4.1387**

Supplementary Figure S3: ***RT^2 Profiler PCR Array analysis of human apoptotic gene expression*****:** Total RNA extracted from LNCaP cells treated with DMSO or BBR (as described in Figs 5A, B) was subjected to cDNA synthesis and analyzed by human apoptosis PCR Array (PAHS-012Z). List above showing the genes with a fold change of >2 (and a *P*-value of <0.05) with some of the highly upregulated genes marked in red and the downregulated gene marked in green. **Gene Abbreviations: CASP5,** Caspase 5, apoptosis-related cysteine peptidase**; GADD45A,** Growth arrest and DNA-damage-inducible, alpha**; HRK,** Harakiri, BCL2 interacting protein (contains only BH3 domain)**; TNFRSF10B,** Tumor necrosis factor receptor superfamily, member 10b**; TNFSF8,** Tumor necrosis factor (ligand) superfamily, member 8**; TNFRSF1B,** Tumor necrosis factor receptor superfamily, member 1B.

Supplementary Table S1

Table S1: Primers Used for qPCR analysis

Primers	Forward	Reverse
hHRK	5’ CAGGCGGAACTTGTAGGAAC	5’ CCAGCTTTCTCCAAGGACAC
hDR4	5’ GGGTCCACAAGACCTTCAAGT	5’ GACACAACTCTCCCAAAGGG
hDR5	5’ AGACCCTTGTGCTCGTTGTC	5’ TTGTTGGGTGATCAGAGCAG
h18S	5’ GGCCCTGTAATTGGAATGAGTC	5’ CCAAGATCCAACTACGAGCTT

Supplementary Table S2

Table S2: Data for qPCR analysis

Target	Time (h)	DMSO (N=3) (Mean ± S.D.)	50µM BBR (N=3) (Mean ± S.D.)	*P* value
hHRK	2	0.001668 ± 0.0004377	0.001215 ± 0.0004963	ns
	4	0.003228 ± 0.0005515	0.004272 ± 0.001459	ns
	8	0.002386 ± 0.0003599	0.004928 ±0.0007747	0.0409*
	16	0.001492 ± 0.0005145	0.02853 ± 0.001838	0.0001***
hDR4	2	0.001756 ± 0.0001083	0.002801 ± 0.001213	ns
	4	0.002256 ± 0.0004200	0.001655 ± 0.0001928	ns
	8	0.002431 ± 0.0005276	0.004455 ± 0.001487	ns
	16	0.001925 ± 0.0002436	0.003780 ± 0.0005128	0.0309*
hDR5	2	0.01537 ± 0.004226	0.02412 ± 0.004059	ns
	4	0.02732 ± 0.01297	0.02763 ± 0.003707	ns
	8	0.01877 ± 0.003450	0.04316 ± 0.0008783	0.0024**
	16	0.01498 ±0.004821	0.04465 ± 0.002123	0.0049**

Note: Significant differences in each treatment between DMSO and BBR, determined by *t*-test, are indicated: ns, $P > 0.05$, * $P \leq 0.05$, ** $P \leq 0.01$, *** $P \leq 0.001$.

Supplementary Figs S4-S7- Full length uncropped blots used in this study

Supplementary Fig S4

<u>Figure- 2A</u>

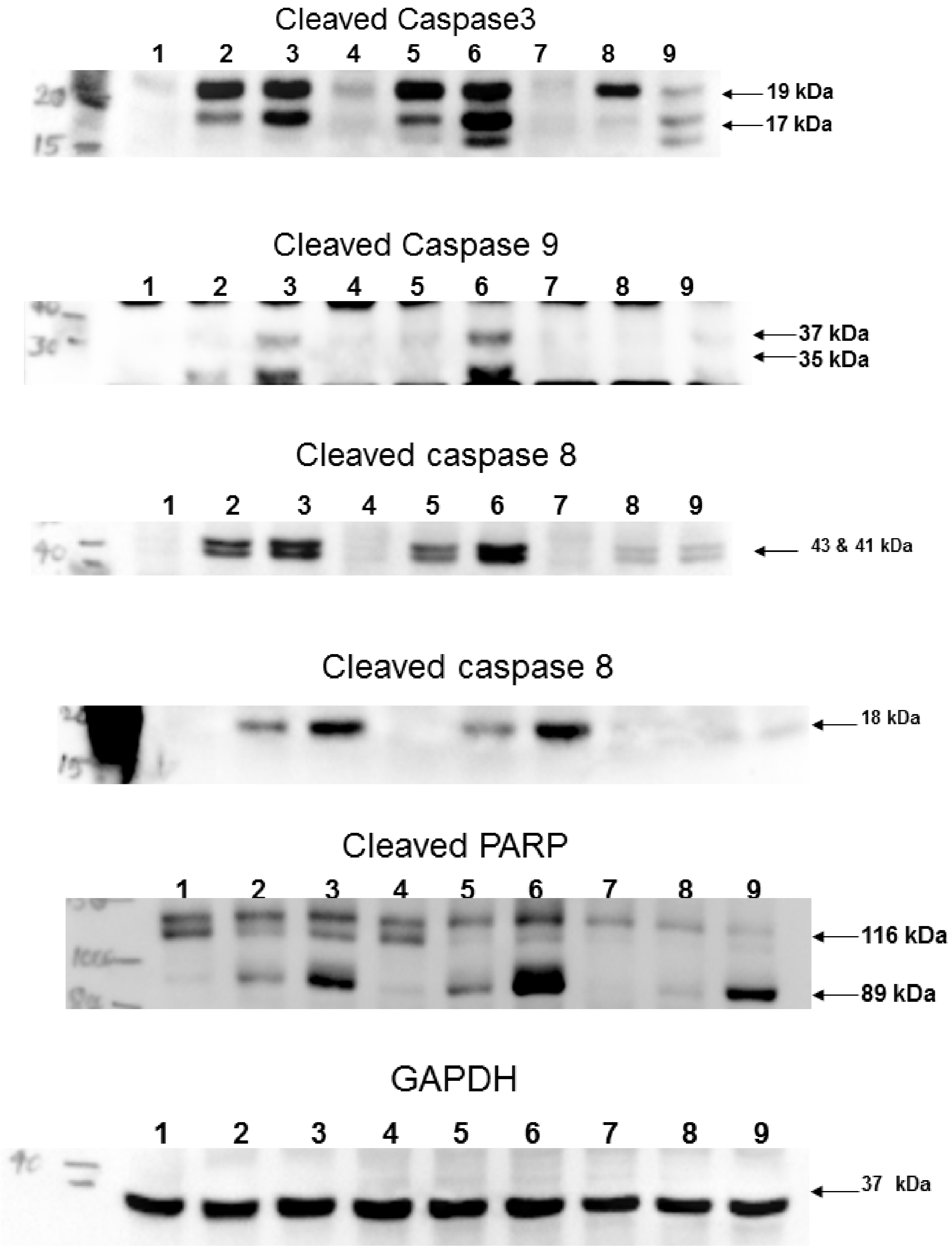

Figure- 2B

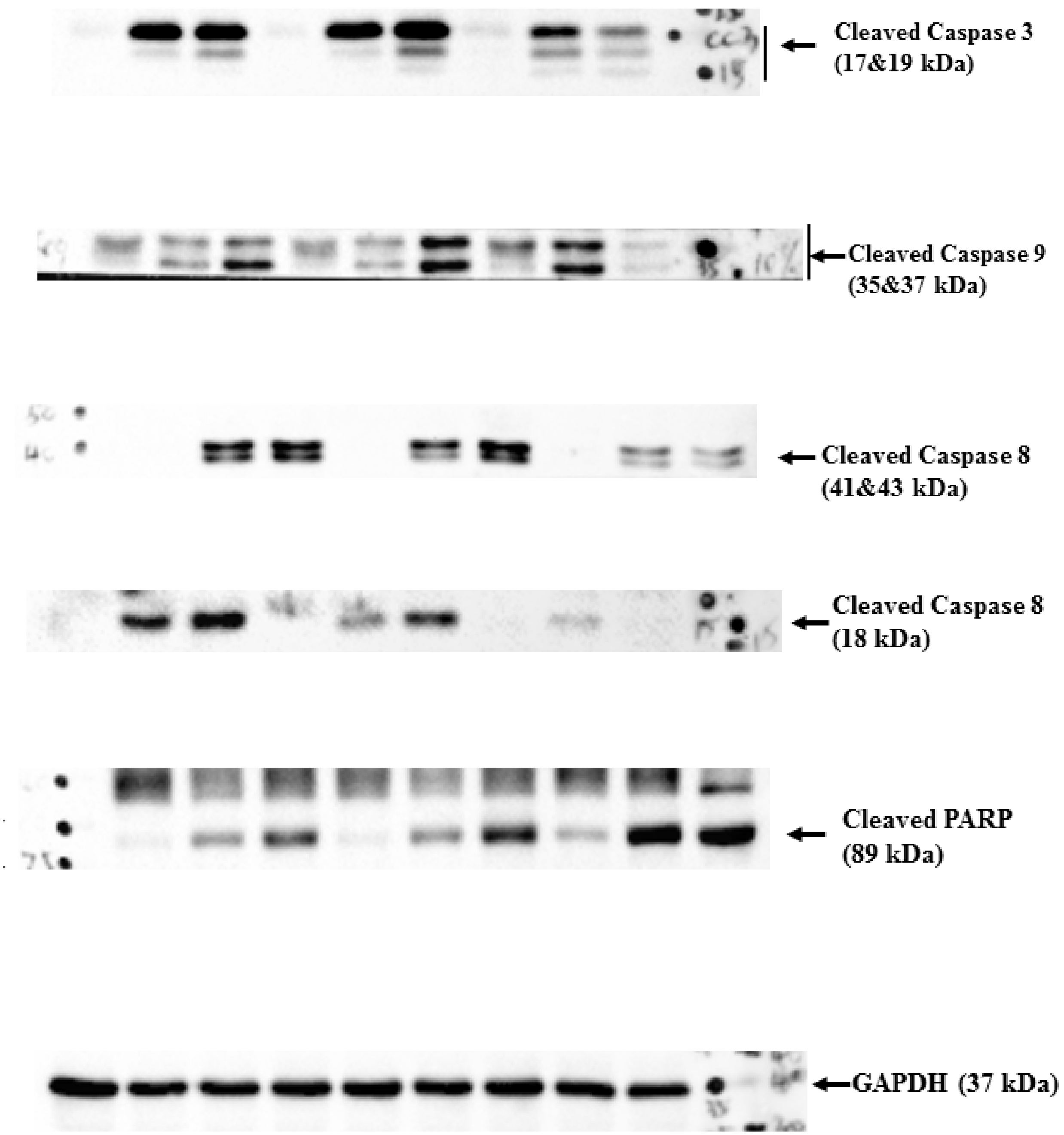
Cleaved Caspase 3
(17&19 kDa)
Cleaved Caspase 9
(35&37 kDa)
Cleaved Caspase 8
(41&43 kDa)
Cleaved Caspase 8
(18 kDa)
Cleaved PARP
(89 kDa)
GAPDH (37 kDa)

Figure- 2C

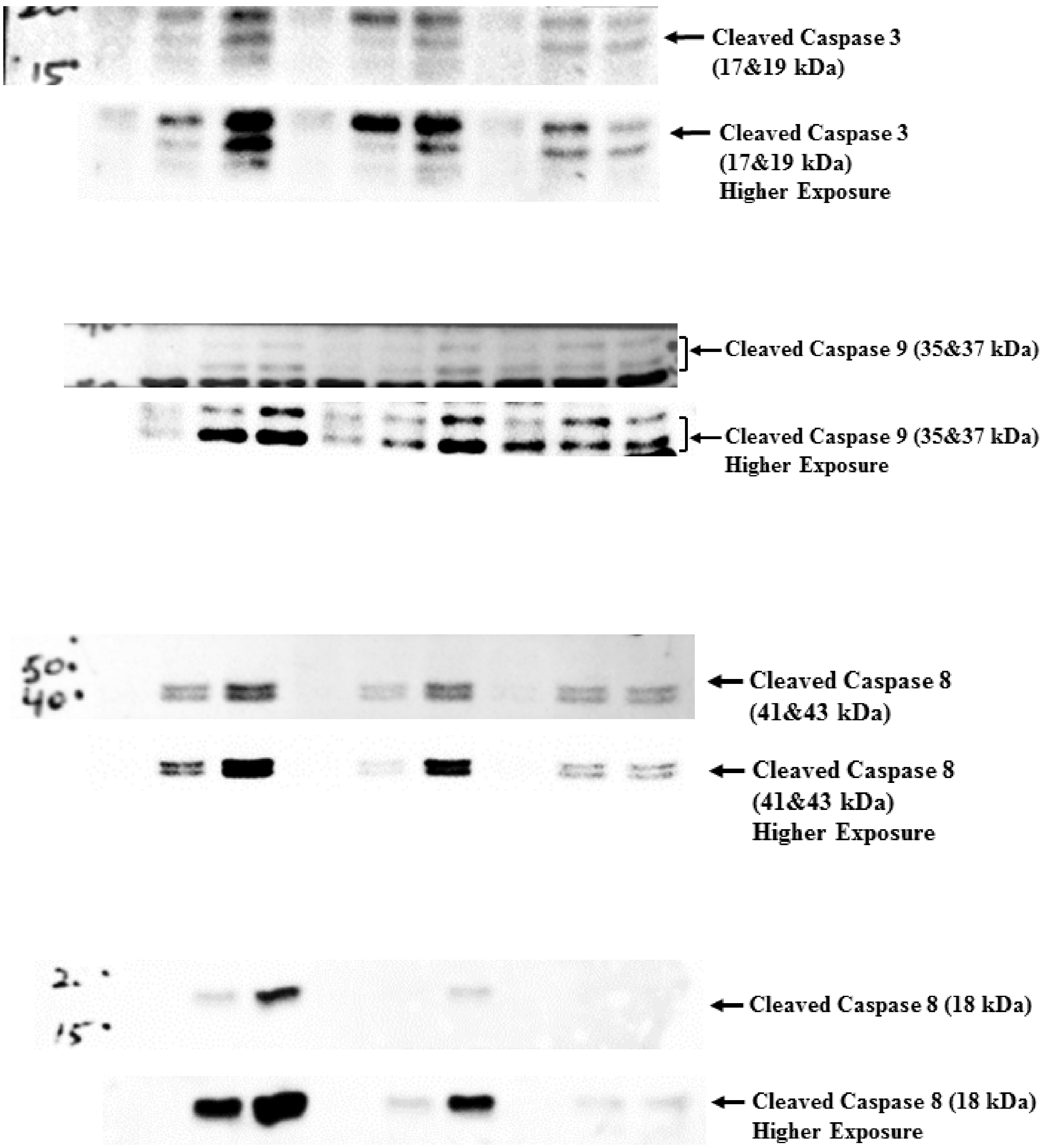

Cleaved Caspase 3
(17&19 kDa)
Cleaved Caspase 3
(17&19 kDa)
Higher Exposure
Cleaved Caspase 9 (35&37 kDa)
Cleaved Caspase 9 (35&37 kDa)
Higher Exposure
Cleaved Caspase 8
(41&43 kDa)
Cleaved Caspase 8
(41&43 kDa)
Higher Exposure
Cleaved Caspase 8 (18 kDa)
Cleaved Caspase 8 (18 kDa)
Higher Exposure

Figure- 2C contd.

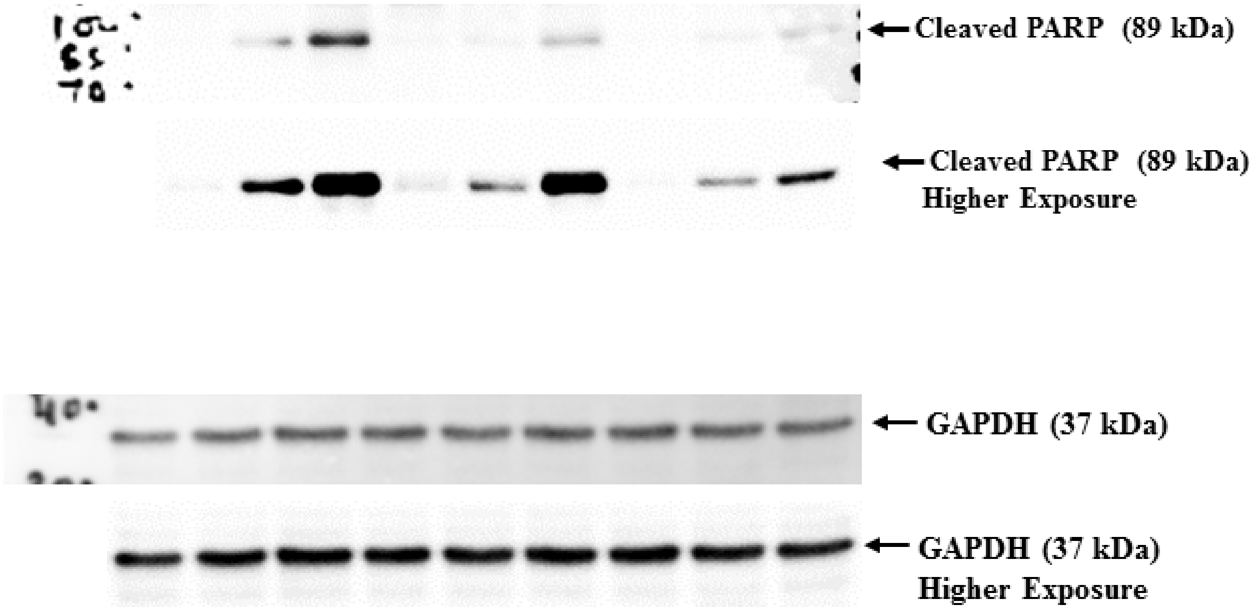

Figure- 2D

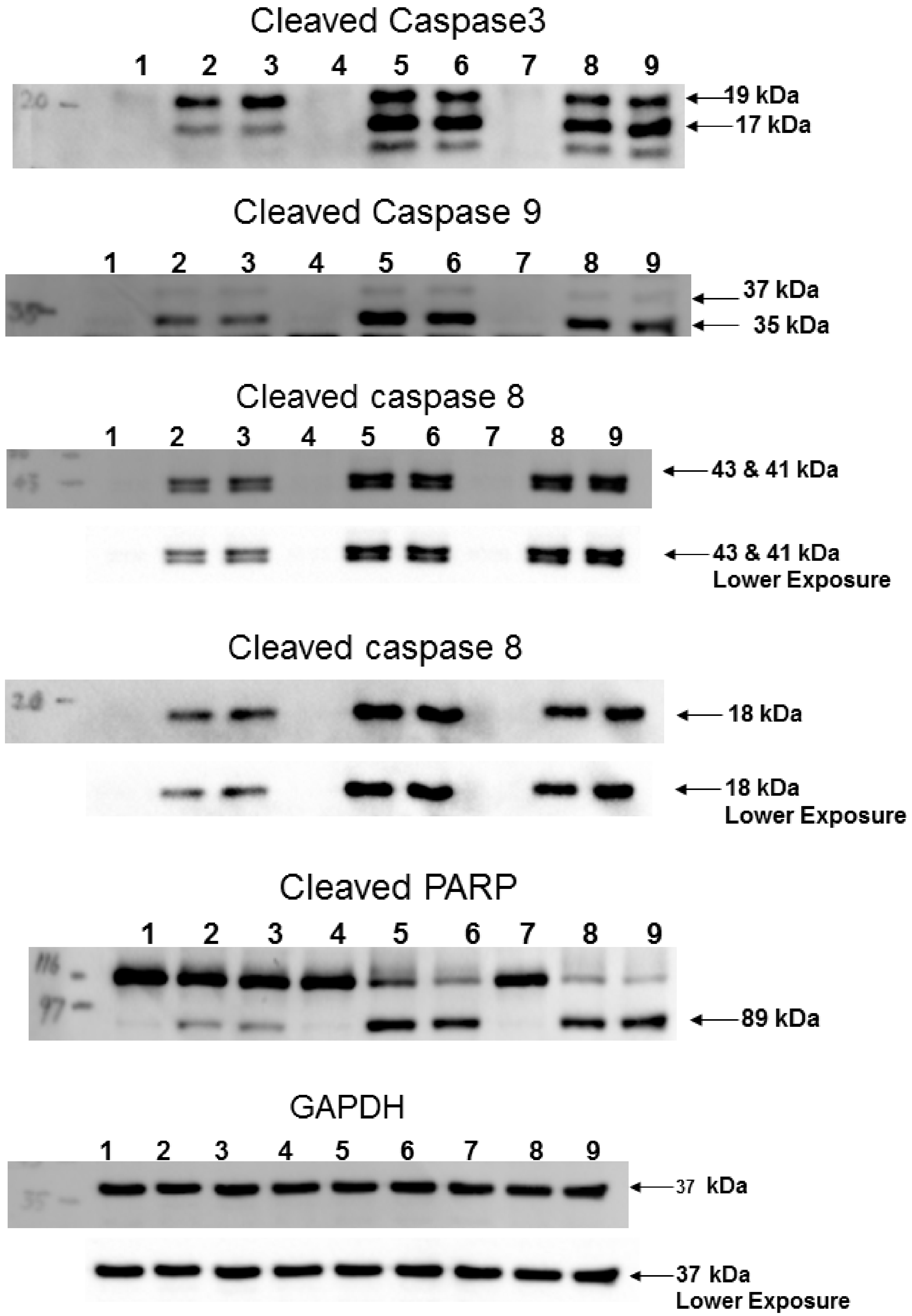

Cleaved Caspase3
1 2 3 4 5 6 7 8 9
19 kDa
17 kDa
Cleaved Caspase 9
1 2 3 4 5 6 7 8 9
37 kDa
35 kDa
Cleaved caspase 8
1 2 3 4 5 6 7 8 9
43 & 41 kDa
43 & 41 kDa
Lower Exposure
Cleaved caspase 8
18 kDa
18 kDa
Lower Exposure
Cleaved PARP
1 2 3 4 5 6 7 8 9
89 kDa
GAPDH
1 2 3 4 5 6 7 8 9
37 kDa
37 kDa
Lower Exposure

Supplementary Fig S5

<u>Figure- 3A</u>

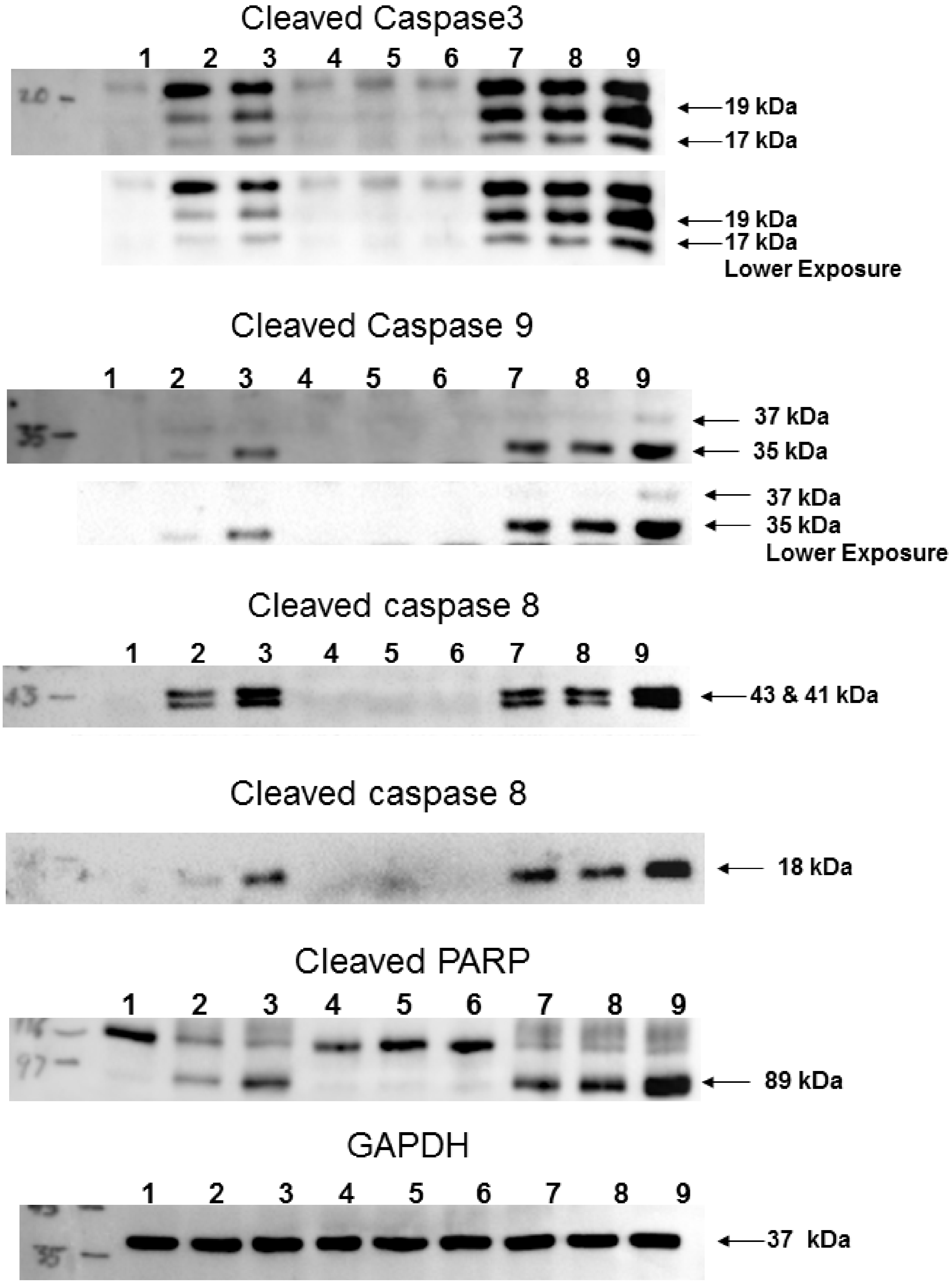

Figure- 3B

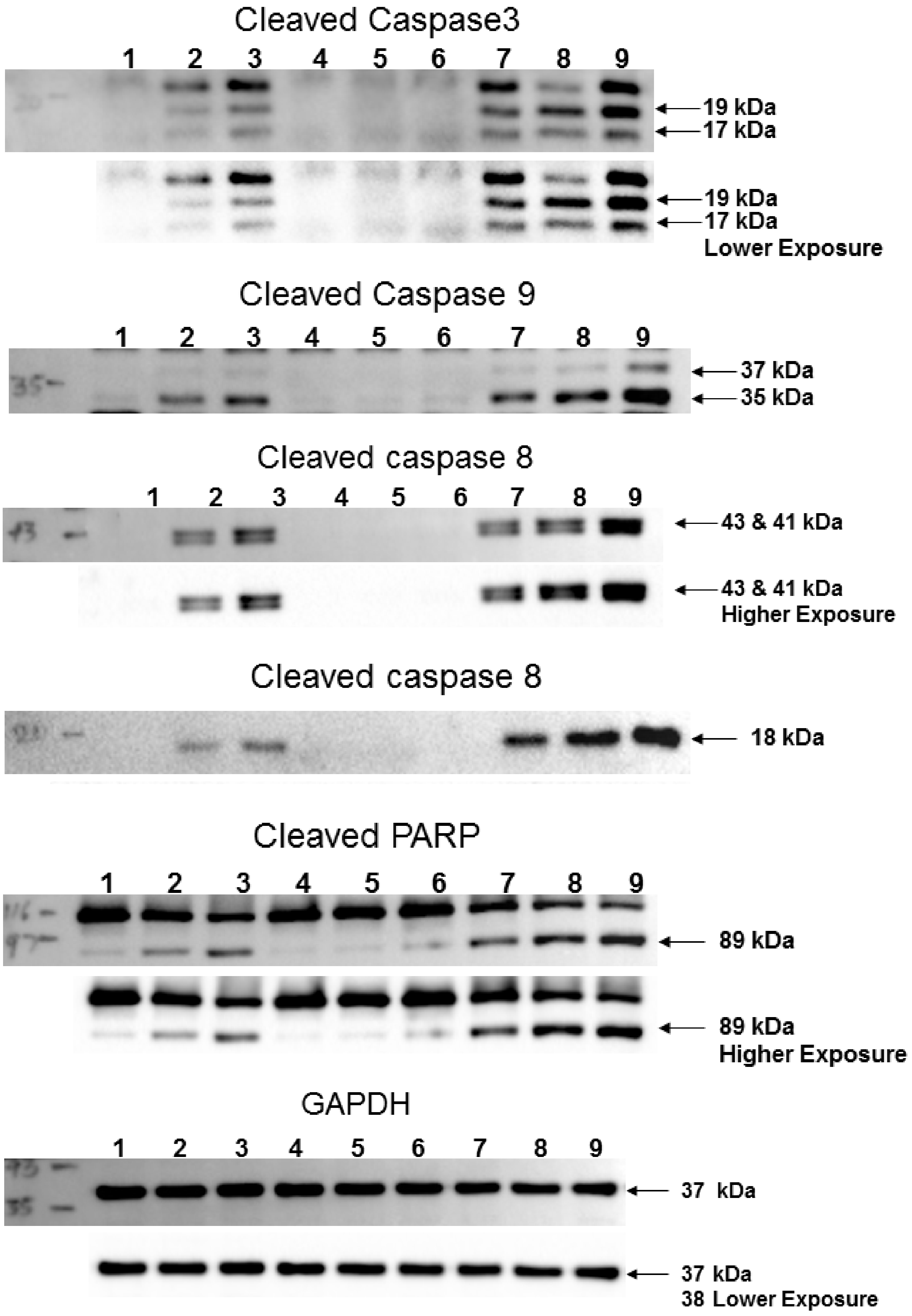

Cleaved Caspase3
1 2 3 4 5 6 7 8 9
19 kDa
17 kDa
19 kDa
17 kDa
Lower Exposure
Cleaved Caspase 9
1 2 3 4 5 6 7 8 9
37 kDa
35 kDa
Cleaved caspase 8
1 2 3 4 5 6 7 8 9
43 & 41 kDa
43 & 41 kDa
Higher Exposure
Cleaved caspase 8
18 kDa
Cleaved PARP
1 2 3 4 5 6 7 8 9
89 kDa
89 kDa
Higher Exposure
GAPDH
1 2 3 4 5 6 7 8 9
37 kDa
37 kDa
38 Lower Exposure

Supplementary Fig S6

Figure- 4A

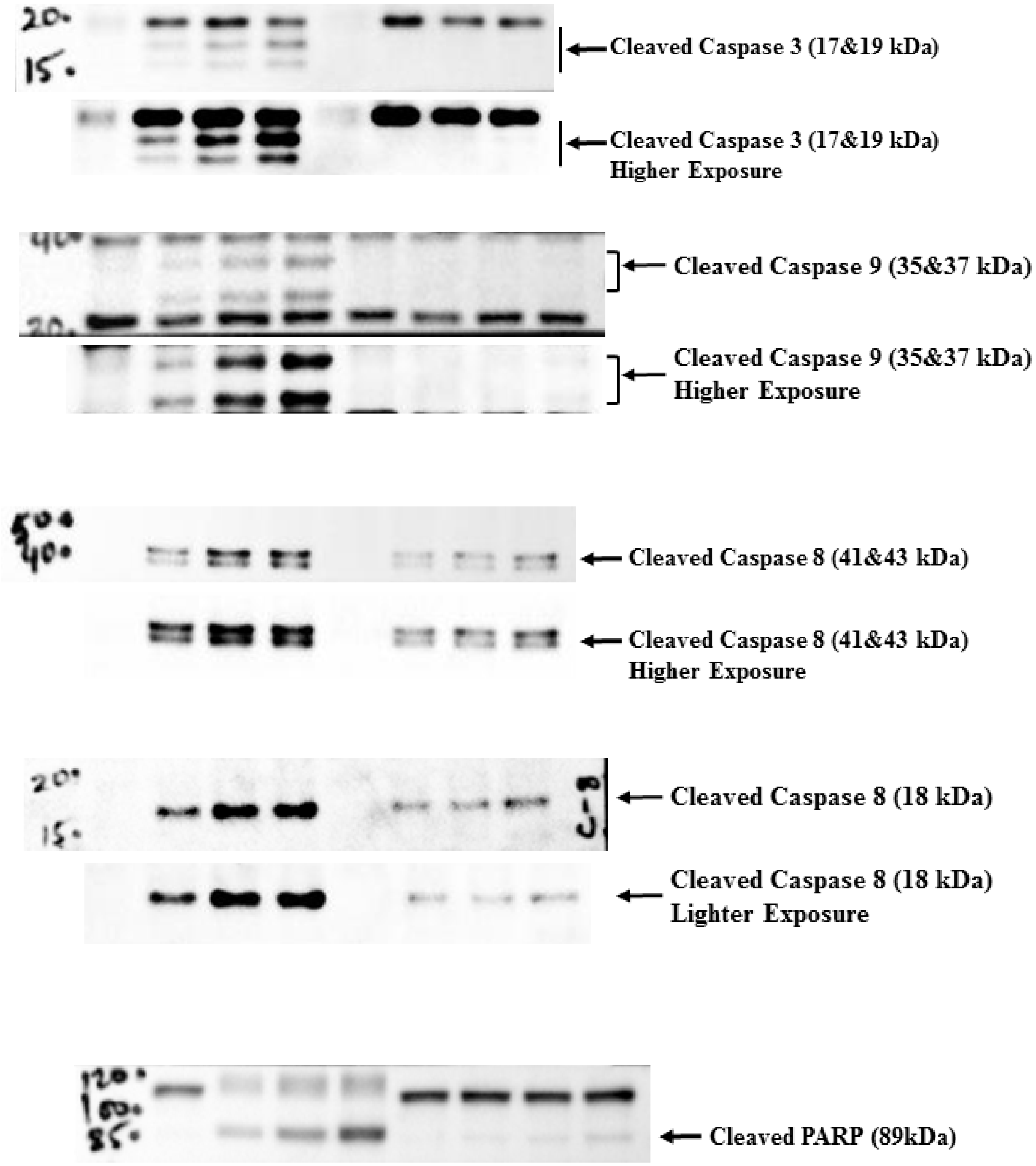

Figure- 4A contd.

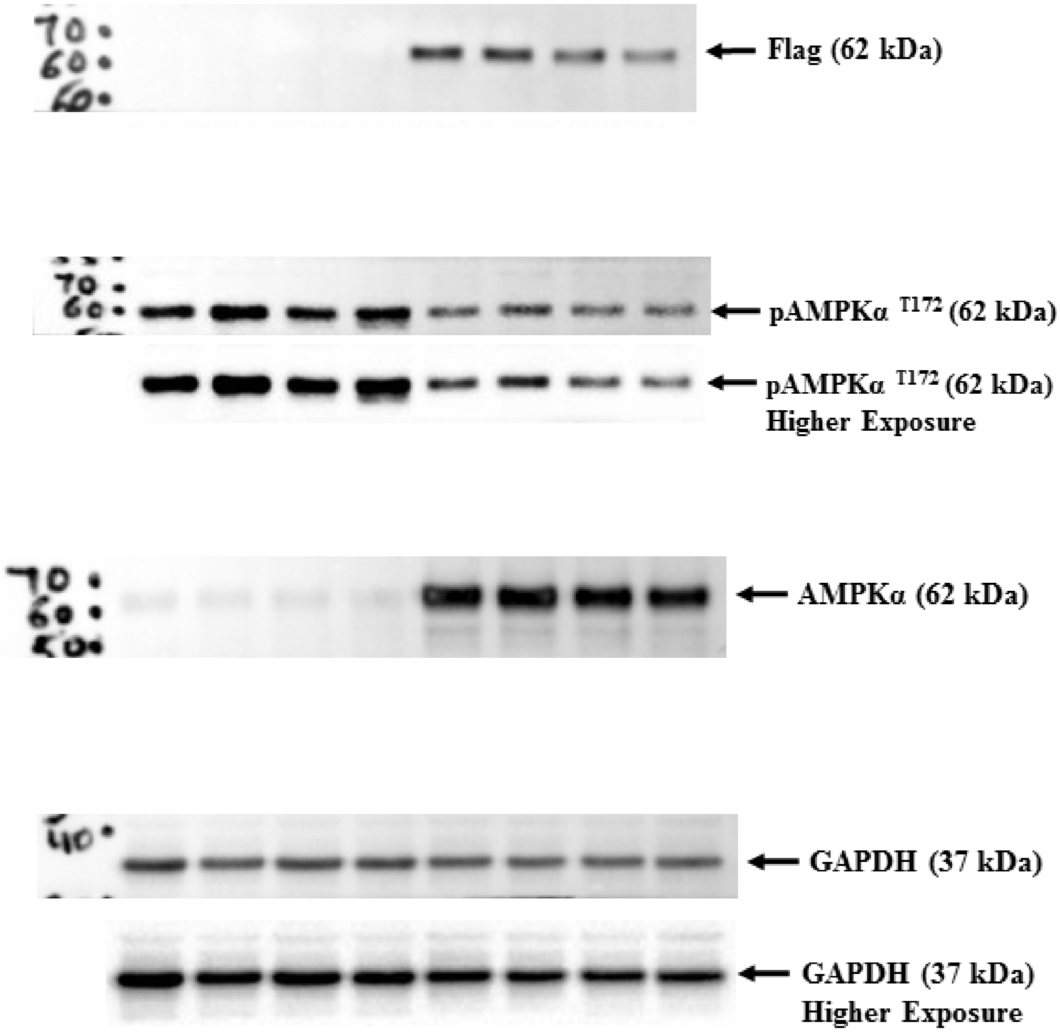

Figure- 4B

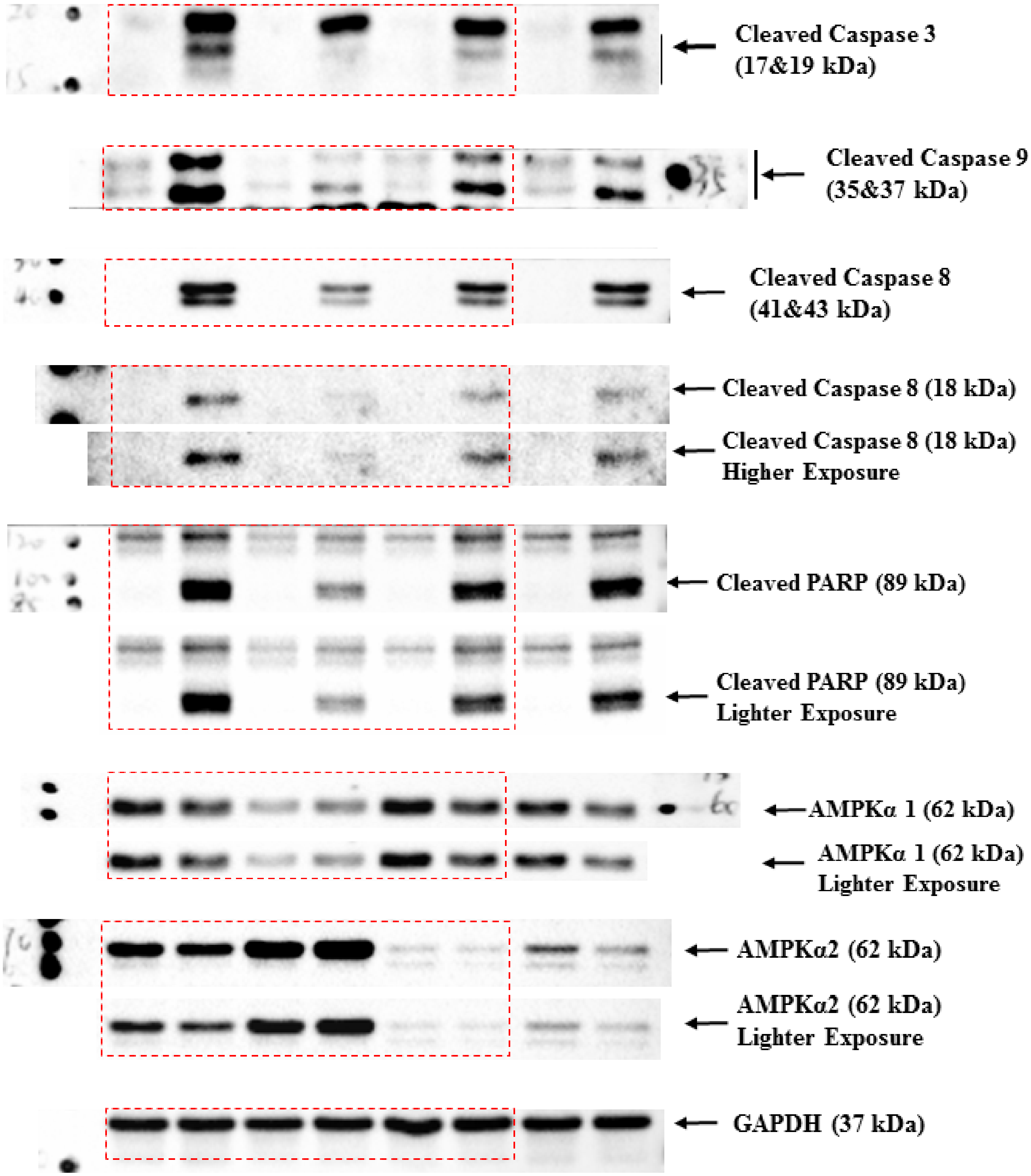

Cleaved Caspase 3
(17&19 kDa)
Cleaved Caspase 9
(35&37 kDa)
Cleaved Caspase 8
(41&43 kDa)
Cleaved Caspase 8 (18 kDa)
Cleaved Caspase 8 (18 kDa)
Higher Exposure
Cleaved PARP (89 kDa)
Cleaved PARP (89 kDa)
Lighter Exposure
AMPKα 1 (62 kDa)
AMPKα 1 (62 kDa)
Lighter Exposure
AMPKα2 (62 kDa)
AMPKα2 (62 kDa)
Lighter Exposure
GAPDH (37 kDa)

Supplementary Fig S7

Figure- 7A

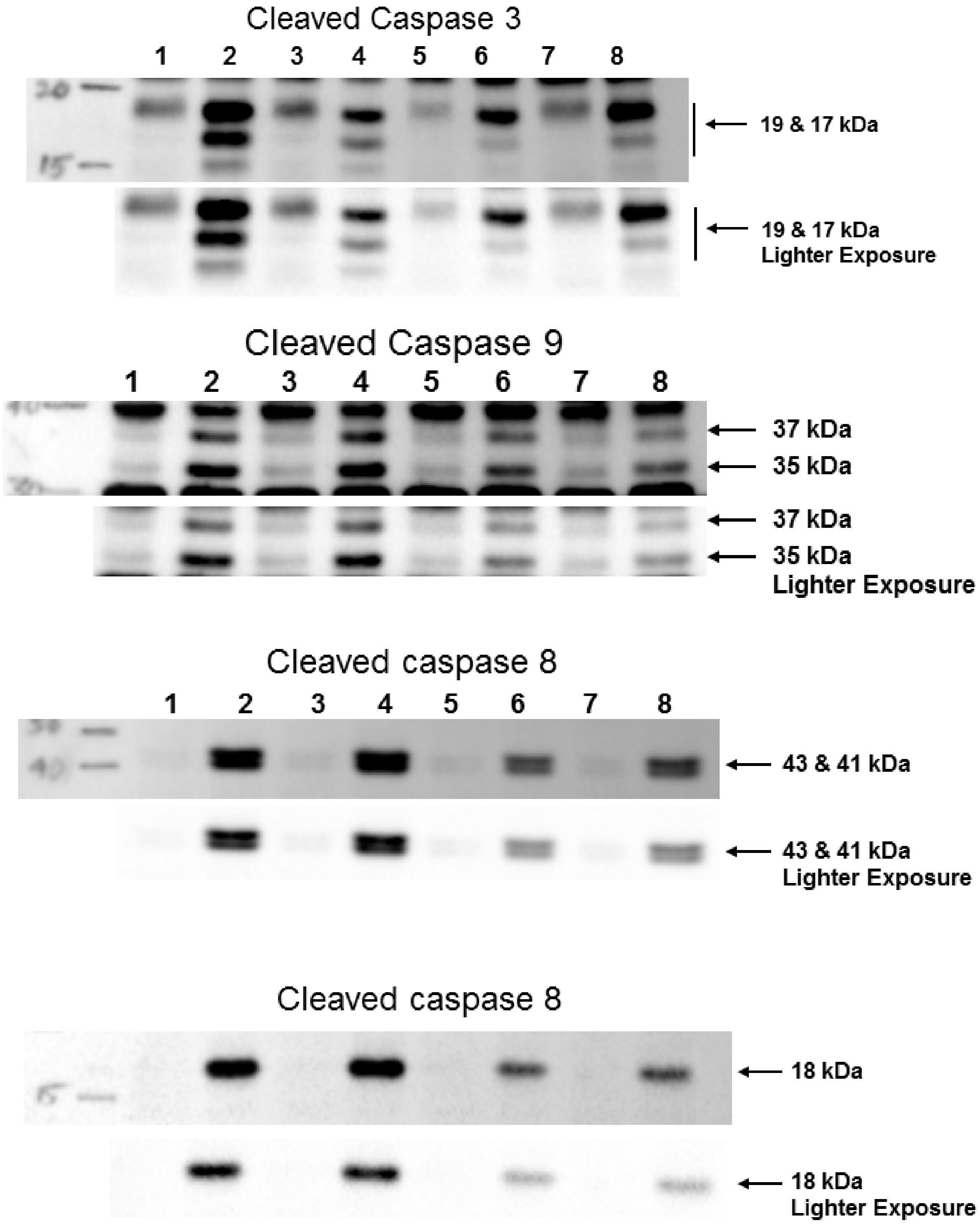

Figure- 7A contd.

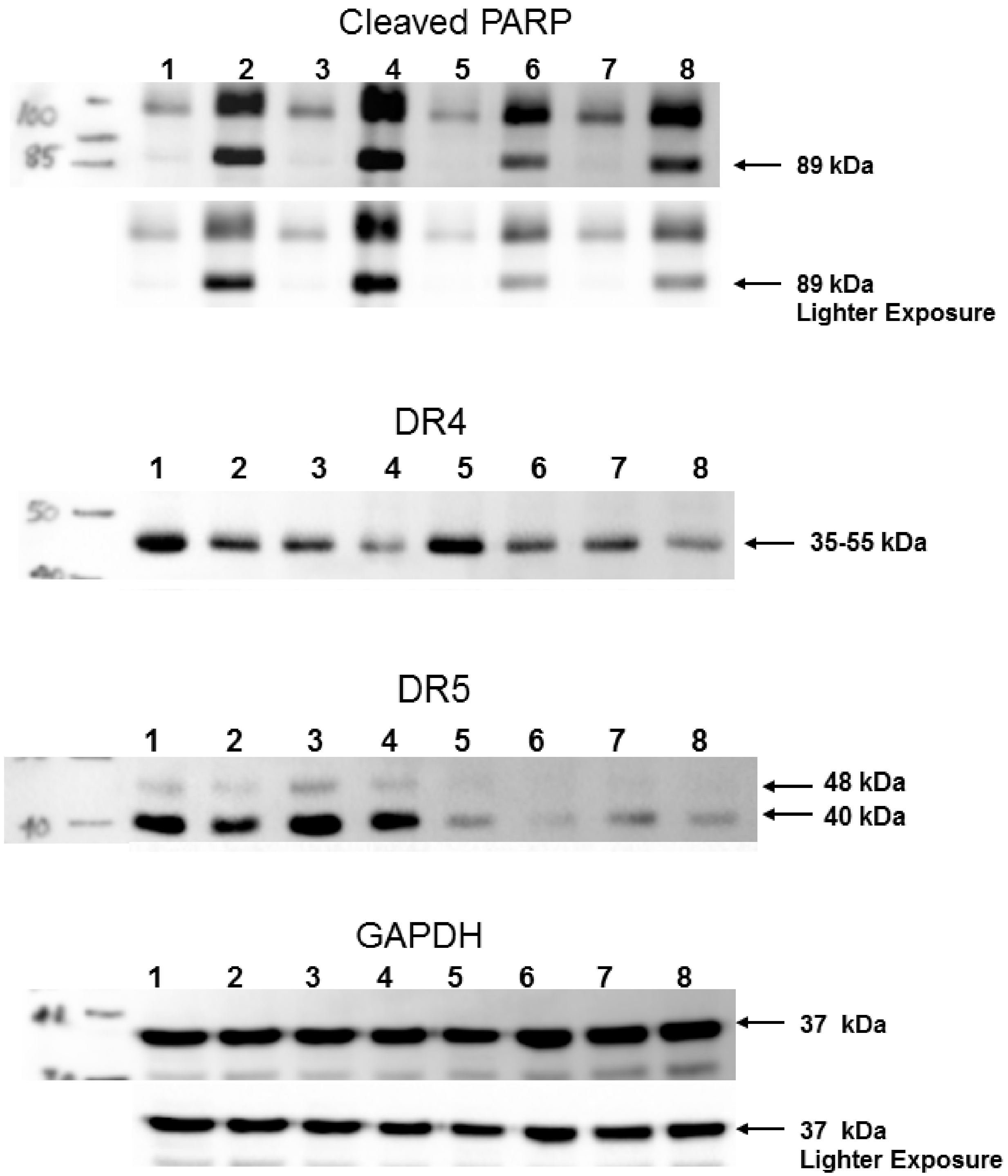

Figure- 7B

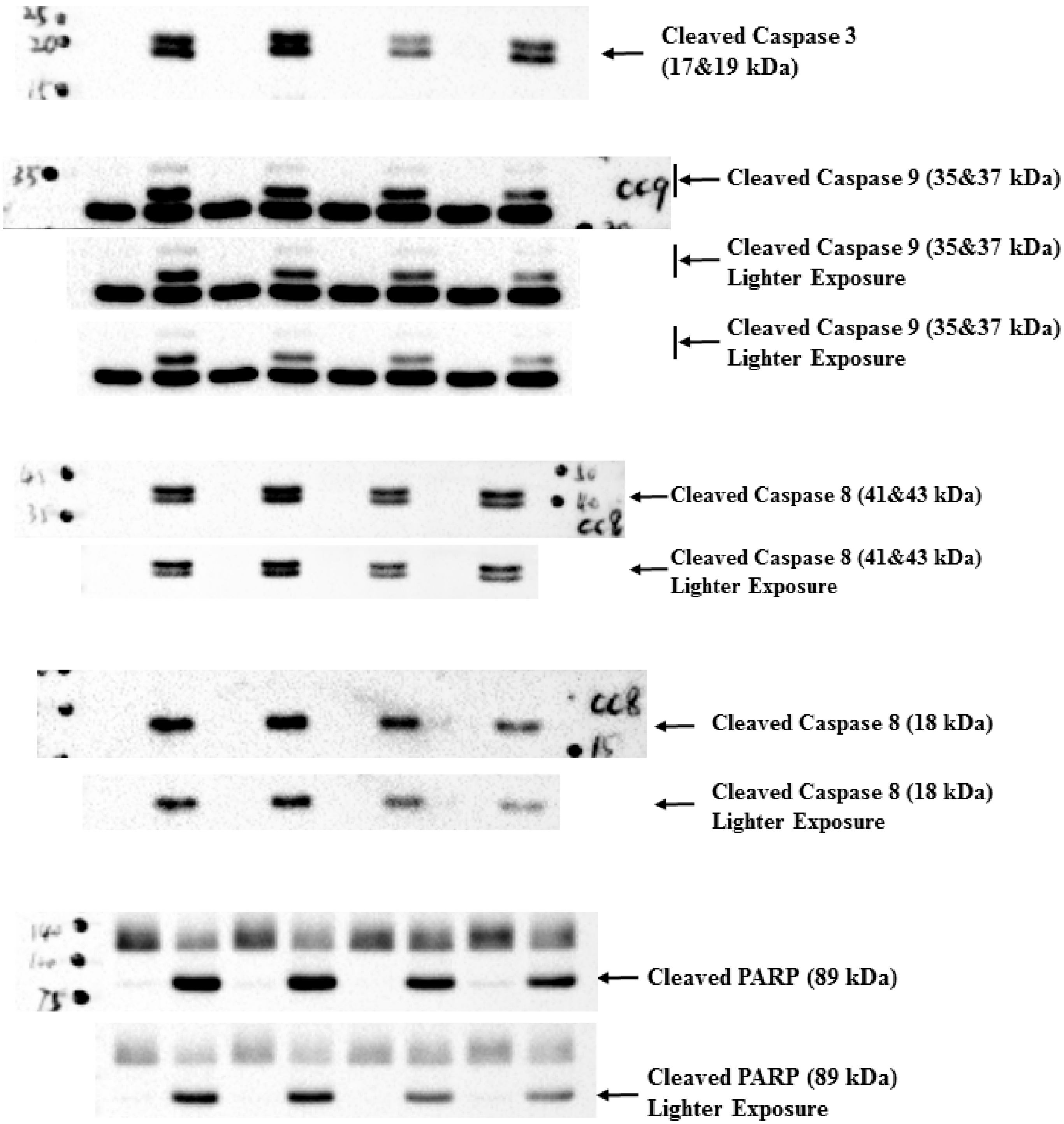
Cleaved Caspase 3 (17&19 kDa)
Cleaved Caspase 9 (35&37 kDa)
Cleaved Caspase 9 (35&37 kDa) Lighter Exposure
Cleaved Caspase 9 (35&37 kDa) Lighter Exposure
Cleaved Caspase 8 (41&43 kDa)
Cleaved Caspase 8 (41&43 kDa) Lighter Exposure
Cleaved Caspase 8 (18 kDa)
Cleaved Caspase 8 (18 kDa) Lighter Exposure
Cleaved PARP (89 kDa)
Cleaved PARP (89 kDa) Lighter Exposure

Figure- 7B contd.

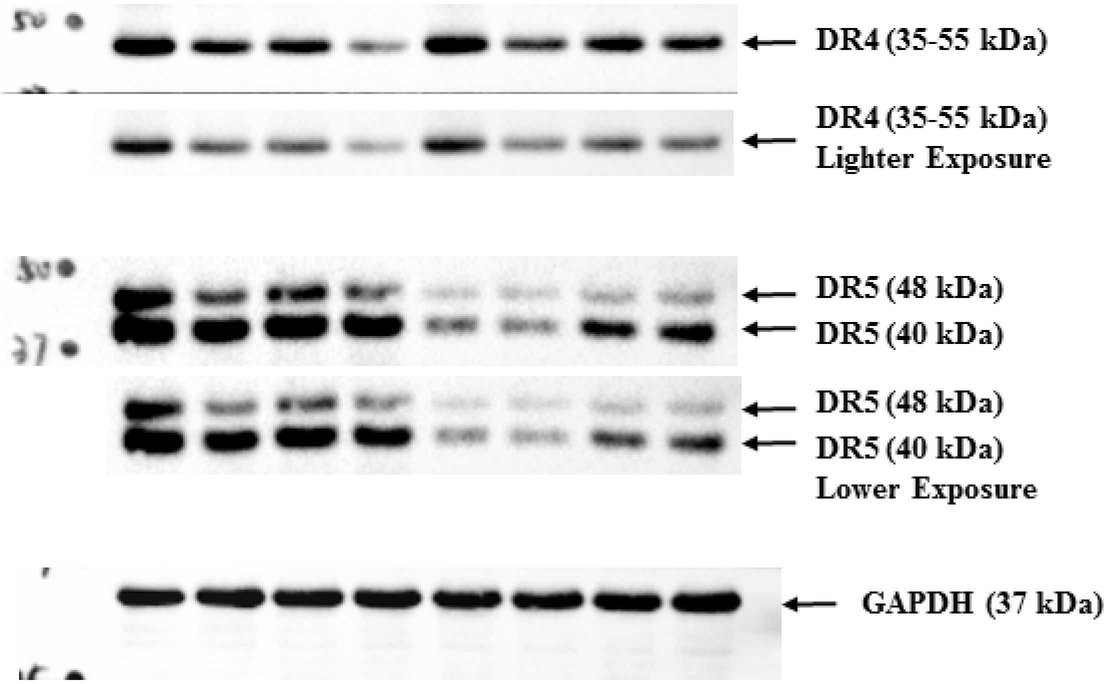

Supplement: Supplementary file 1 — Supplementary Information [file 41598_2018_23780_MOESM1_ESM.pdf]
